# Playing Analog Games Is Associated With Reduced Declines in Cognitive Function: A 68-Year Longitudinal Cohort Study

**DOI:** 10.1093/geronb/gbz149

**Published:** 2019-11-18

**Authors:** Drew M Altschul, Ian J Deary

**Affiliations:** 1 Department of Psychology, The University of Edinburgh, UK; 2 Mental Health Data Science Scotland, Royal Edinburgh Hospital, Edinburgh, UK; 3 Centre for Cognitive Ageing and Cognitive Epidemiology, The University of Edinburgh, UK

**Keywords:** Cognition, Cognitive reserve, Games, Intellectual functioning, Longitudinal change

## Abstract

**Objectives:**

Playing analog games may be associated with better cognitive function but, to date, these studies have not had extensive longitudinal follow-up. Our goal was to examine the association between playing games and change in cognitive function from age 11 to age 70, and from age 70 to 79.

**Method:**

Participants were 1,091 nonclinical, independent, community-dwelling individuals all born in 1936 and residing in Scotland. General cognitive function was assessed at ages 11 and 70, and hierarchical domains were assessed at ages 70, 73, 76, and 79 using a comprehensive cognitive battery of 14 tests. Games playing behaviors were assessed at ages 70 and 76. All models controlled for early life cognitive function, education, social class, sex, activity levels, and health issues. All analyses were preregistered.

**Results:**

Higher frequency of playing games was associated with higher cognitive function at age 70, controlling for age 11 cognitive function, and the majority of this association could not be explained by control variables. Playing more games was also associated with less general cognitive decline from age 70 to age 79, and in particularly, less decline in memory ability. Increased games playing between 70 and 76 was associated with less decline in cognitive speed.

**Discussion:**

Playing games were associated with less relative cognitive decline from age 11 to age 70, and less cognitive decline from age 70 to 79. Controlling for age 11 cognitive function and other confounders, these findings suggest that playing more games is linked to reduced lifetime decline in cognitive function.

Living an intellectually engaged life appears to have long-term benefits for maintaining one’s cognitive functions (Staff, Hogan, Williams, & Whalley, 2018). Specific attributes such as bilingualism (Bak, Nissan, Allerhand, & Deary, 2014) and working an intellectually-demanding job (Smart, Gow, & Deary, 2014), as well as general measures like typical intellectual engagement (Staff et al., 2018; Von Stumm & Deary, 2012) have all been associated with having better cognitive functions later in life. Successful cognitive aging, that is, slower cognitive decline in middle and older age, is of major interest in the fields of public health and geriatric medicine, and for aging individuals themselves (Corley, Cox, & Deary, 2018). Cognitive decline is associated with poorer mental and physical health outcomes, such as diabetes (Altschul, Starr, & Deary, 2018) and depression (Aichele et al., 2018). Moreover, cognitive decline is also related to increased health care costs (Brayne, 2007), poorer quality of life (Plassman, Williams, Burke, Holsinger, & Benjamin, 2010), as well as impaired decision making and everyday functional abilities (Tucker-Drob, 2011).

In the search for interventions that could reduce the rate of cognitive decline, brain training, and other digital cognitive games have come under particular and extensive study (Anguera et al., 2013; Ball et al., 2002; Ngandu et al., 2015). But, whether digital cognitive games and so-called brain training have a protective effect on cognitive functions is controversial (Kelly et al., 2014; Kueider, Parisi, Gross, & Rebok, 2012; Lampit, Hallock, & Valenzuela, 2014; Simons et al., 2016); effects are often not robust (Lampit et al., 2014) or do not transfer beyond the context of the games (Melby-Lervåg, Redick, & Hulme, 2016), and positive effects that are demonstrated do not always last (Zhang & Kaufman, 2016). However, evidence also suggests that more traditional, analog games such as board games (Dartigues et al., 2013), cards (Kuo, Huang, & Yeh, 2018), crosswords and Sudoku (Ferreira, Owen, Mohan, Corbett, & Ballard, 2015) all protect somewhat against cognitive decline.

Nevertheless, previous studies of analog games have had limitations. In cross-sectional analyses, more game playing was associated with higher cognitive functions (Ferreira et al., 2015; Brooker et al., 2018), but these studies could not examine cognitive change over time, or control for many confounding effects. Crucially, they do not test for confounding by cognitive ability from earlier in life. A longitudinal examination of the association between board game playing and dementia found a protective effect among more regular players, but this effect disappeared when mental health was controlled for (Dartigues et al., 2013). A lone randomized controlled trial found evidence that cognitively stimulating card and board games improve executive functions (Kuo et al., 2018), but the trial was limited by small sample size and brief follow-up times; moreover, they found no associations between playing games and reasoning or episodic memory abilities. Overall, evidence for positive effects of playing analog games is no stronger than for digital games.

In the present study, we use data from participants in the Lothian Birth Cohort of 1936 (LBC1936) to address the question: Does playing analog games protect against cognitive decline in older age? The LBC1936 are unusually valuable for seeking an answer; they provide historical data on early-life cognitive function and other life course variables, as well as repeated and detailed measurements of several cognitive functions in later life, analog games playing habits, and potential confounders. We preregistered hypotheses and analyses that evaluated game playing’s relationship with: (a) change In general, cognitive function from age 11 to 70 years; and (b) its associations with change in four specific cognitive subdomains measured on four occasions between age 70 and 79: visuospatial or fluid ability, processing speed, memory, and crystallized ability.

## Method

### Study Sample

The Lothian Birth Cohort 1936 (LBC1936) is a community-dwelling sample of 1,091 initially healthy individuals. All were born in 1936 and were at school in Scotland on 4 June 1947, when most took part in a group-administered intelligence test: the Moray House Test (MHT) No. 12. They were followed up in four waves of one-to-one cognitive and health testing between 2004 and 2017, at mean ages 70 (*N* = 1,091), 73 (*N* = 866), 76 (*N =* 697), and 79 (*N =* 550) years. Further details on the background, recruitment, attrition, and data collection procedures are available (Taylor, Pattie, & Deary, 2018). Participants provided written informed consent. Ethics permissions were obtained from the Multicentre Research Ethics Committee for Scotland (Wave 1, MREC/01/0/56), the Lothian Research Ethics Committee (Wave 1, LREC/2003/2/29), and the Scotland a Research Ethics Committee (Waves 2–4, 07/MRE00/58).

### Cognitive Functions

The MHT is a broad cognitive ability test that includes word classification, proverbs, spatial items, and arithmetic. The test correlated about 0.8 at age 11 years with the Terman Merrill revision of the Stanford–Binet test, providing concurrent validity (Deary, Whalley, & Starr, 2009). At Wave 1 of follow-up, in older age (mean age 70 years), participants repeated the MHT where concurrent validity was independently established (Deary, Johnson, & Starr, 2010).

In four waves of data collected in older age, 14 individually administered cognitive tests were used to assess three subdomains of cognitive function that decline with age: fluid, processing speed, and memory ability, as well as crystallized ability. A general factor of cognitive function was also hierarchically modeled from the subdomains. The tests are fully described and referenced in an open-access protocol article (Taylor et al., 2018), and the details of how the tests are associated with different domains is described in Supplementary Appendix 1.

Eleven participants were excluded from our analyses of cognitive change from age 11 to 70 because they either had a history of dementia or scored less than 24 on the MMSE at age 70. Thirty-seven participants were excluded from our analyses of change from age 70 to 79 because they had a history of dementia or scored less than 24 on the MMSE at any point between age 70 and 79.

### Playing Games

As part of a larger questionnaire on social and physical activities (Gow, Corley, Starr, & Deary, 2012), LBC1936 participants indicated in Wave 1 at age 70 how often they generally engaged in each activity. The particular item investigated here was “Playing games (like cards, chess, bingo, or crosswords).” Participants endorsed one of “Every day or about every day,” “Several times a week,” “Several times a month,” “Several times a year,” or “Less than once a year/never.” Responses were assigned ordinal values of 1–5 with, “Every day or about every day” registering as a 5. Using a repeated assessment of this question in Wave 3 at age 76, we also created binary and ordinal variables, the first indicating which individuals reported any increase in game playing frequency between age 70 and 76, and the second indicating the degree of change in games playing frequency between 70 and 76.

### Covariates

Three sets of variables were evaluated as potential confounders. The first set defined our sociodemographic covariates: sex, years of education, and the participant’s adult social class. The second set of covariates consisted of variables describing other activities, assessed by questionnaire as described previously in this sample by Gow et al. (2012). 984 questionnaires were received at age 70. An aggregate variable representing overall sociointellectual activity was generated using principal components analysis. The complete details of these analyses are available in Supplementary Appendix 2.

The third set of covariates included major medical diagnosis risk factors for cognitive decline. At age 70, participants self-reported if they had history of high blood pressure, stroke, diabetes, or cardiovascular disease. Each was coded as a binary variable, with a 1 indicating that the individual had a history of the disease.

### Statistical Analyses

Unless otherwise noted, all analyses were preregistered on the Open Science Framework (OSF) before the data were requested from the LBC1936 database manager and downloaded—see https://osf.io/wdgm3/. All analyses were carried out in the R statistical programming language (version 3.5.1).

### Playing Games, Cognitive Change from Age 11 and 70, and Confounding by Life Circumstances

Our first set of analyses examined cognitive change between age 11 and 70 using data collected at age 70. These data were detailed health and cognitive testing information at age 70 as well as historical data from each individual’s life. The independent variable (exposure) of interest in our regression models was the ordinal playing games variable, and the other covariates were also included as independent variables. The dependent (outcome) variable was either cognitive function, assessed with the MHT, at age 70 or the change in MHT score from age 11 to 70. Linear regressions were modeled with base R functions.

**Figure 1.**
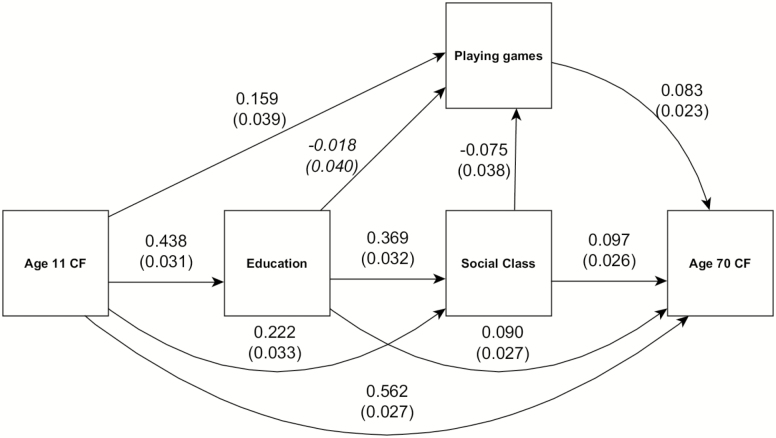
Life course path diagram of the regressions among sociodemographic variables, cognitive functions, and playing games. Arrows indicate direction of the regression paths, with the numbers indicating std β weights and std errors (in parentheses). All paths are significant at *p* < .05, except for the path from education to playing games, printed in italics.

A life course path model was specified after the initial preregistration and data download, but no analysis was carried out prior to preregistration. This approach allowed us to model all downstream associations of age 11 cognitive function, education, and social class (Scarmeas & Stern, 2003), on games playing behavior, age 70 cognitive function, and each other—Figure 1 illustrates the paths modeled in this way; at this stage, the numbers in the figure may be ignored. We also specified a path model wherein we controlled for the confounding effect of these same life course variables, and included other sociodemographic and health variables as controls. This model is fully described and presented in Supplementary Appendix 3. These models were fitted using the “psych” package (version 1.8.10) using the Preacher and Hayes (2004) bootstrap method. Path modeling used the “lavaan” package (version 0.6–3).

### Playing Games and Trajectories of Cognitive Change in the Eighth Decade

The second set of analyses examined contributions to cognitive change between age 70 and 79. This was done using latent growth curve models. Latent growth curve models allowed us to introduce a hypothesis-testing approach to longitudinal, correlational data. The correlational aspect of the data was captured by a known hierarchical model (Altschul et al., 2018; Ritchie et al., 2016) that consisted of a general variable of cognitive function and four subdomains of function beneath it. Latent variables representing each subdomain were specified using their cognitive tests, as mentioned in the *Cognitive Function* section. For example, the crystallized ability latent variable captured the common variance of National Adult Reading Test, Wechsler Test of Adult Reading, and a phonemic verbal fluency test score. Each subdomain captured common variance among its constituent tests in this way, and the general function latent variable captured the common variance among the four subdomains. A representative path model diagram is presented in Supplementary Figure S1.

The longitudinal aspect of the analysis was achieved by specifying latent variables that capture change in the cognition latent variables across the four waves. For each of the general cognitive function and four cognitive subdomain variables, we modeled an intercept (i.e., the baseline performance of each individual) and slope (i.e., trajectory of change between age 70 and 79) latent variables. These latent variables could be analyzed as if they were directly measured variables. Thus, cognitive intercept and cognitive slope were regressed onto the same covariates as used in our analyses of cognitive change from age 11 to 70. We were thereby able to assess whether playing games was associated with the baseline (age 70) level of general cognitive ability or any of the specific cognitive subdomains, and decline in any of these cognitive functions. The cognitive intercepts’ and slopes’ latent variables were regressed on all covariates simultaneously, except for the variables measuring change in playing games. Each of those two variables was added as a dependent covariate in distinct additional growth curve models and only associated with the slope variables of cognitive functions.

Model coefficients were estimated using full information maximum likelihood, that is, the estimation optimizer attempted to use all data from all participants, even individuals who did not complete all waves. Standard errors were bootstrapped from 1,000 bootstrap draws, and *p* values were computed using the χ ^2^ test statistic. For each model, univariate or multivariate, a critical value of *α* < 0.05 was considered significant. All growth curve models were fit using “lavaan.”

## Results

### Playing Games at Age 70 and 76

The study sample is described in Table 1. Frequency of playing analog games at age 70 was generally high, with 320 participants (33% of 961) reporting that they played games every day or nearly every day. The distribution was U-shaped, as the second most frequent category (*n* = 195, i.e., 20%) was the lowest, that is, those participants who played games less than once per year or never.

**Table 1.** Descriptive Statistics for Key Variables in LBC1936

Variable	*n*	Mean	SD
Moray house test scores			
Age 11	1,028	49.00	11.80
Age 70	1,079	64.23	8.80
Education	1,091	10.74	1.13
Social class	1,070	3.60	0.91
Activity score	1,091	0.00	0.99
Playing games (age 70)	961	3.11	1.53
Every day or about every day (5)	320		
Several times a week (4)	167		
Several times a month (3)	161		
Several times a year (2)	118		
Less than once a year/never (1)	195		
Playing games (age 76)	682	3.35	1.50
Every day or about every day (5)	222		
Several times a week (4)	135		
Several times a month (3)	108		
Several times a year (2)	94		
Less than once a year/never (1)	123		
Dichotomous variable	*n*	Reference	
Increase in games playing	626	466 (No)	160 (Yes)
Hypertension history	1,091	658	433
Cardiovascular disease history	1,091	823	268
Diabetes history	1,091	1,000	91
Stroke history	1,091	1,037	54
Sex	1,091	548 (male)	543 (female)

*Note*: Moray house test scores = measures of cognitive function, education = number of years in fulltime education, activity score = component score of 13 activity variables.

At age 76, the responses were similar, with 222 participants (again, 33% of 682) reporting that they played games every day or nearly every day, and the second largest category (*n* = 123, i.e., 18%) reported playing games less than once a year or never. In between, the distribution was similarly U-shaped. However, some individuals did change their games playing habits: 160 increased their frequency of playing games to some degree. Reliability of the item was generally good (*ρ* = .63, *ICC(3,1)* = 0.64), which is consistent with previous work suggesting the individuals’ self-reporting of playing games is accurate (Waris et al., 2019). Reliability is discussed in greater depth in Supplementary Appendix 4.

### Playing Games and Cognitive Function From Age 11 Years to Age 70

**Figure 2.**
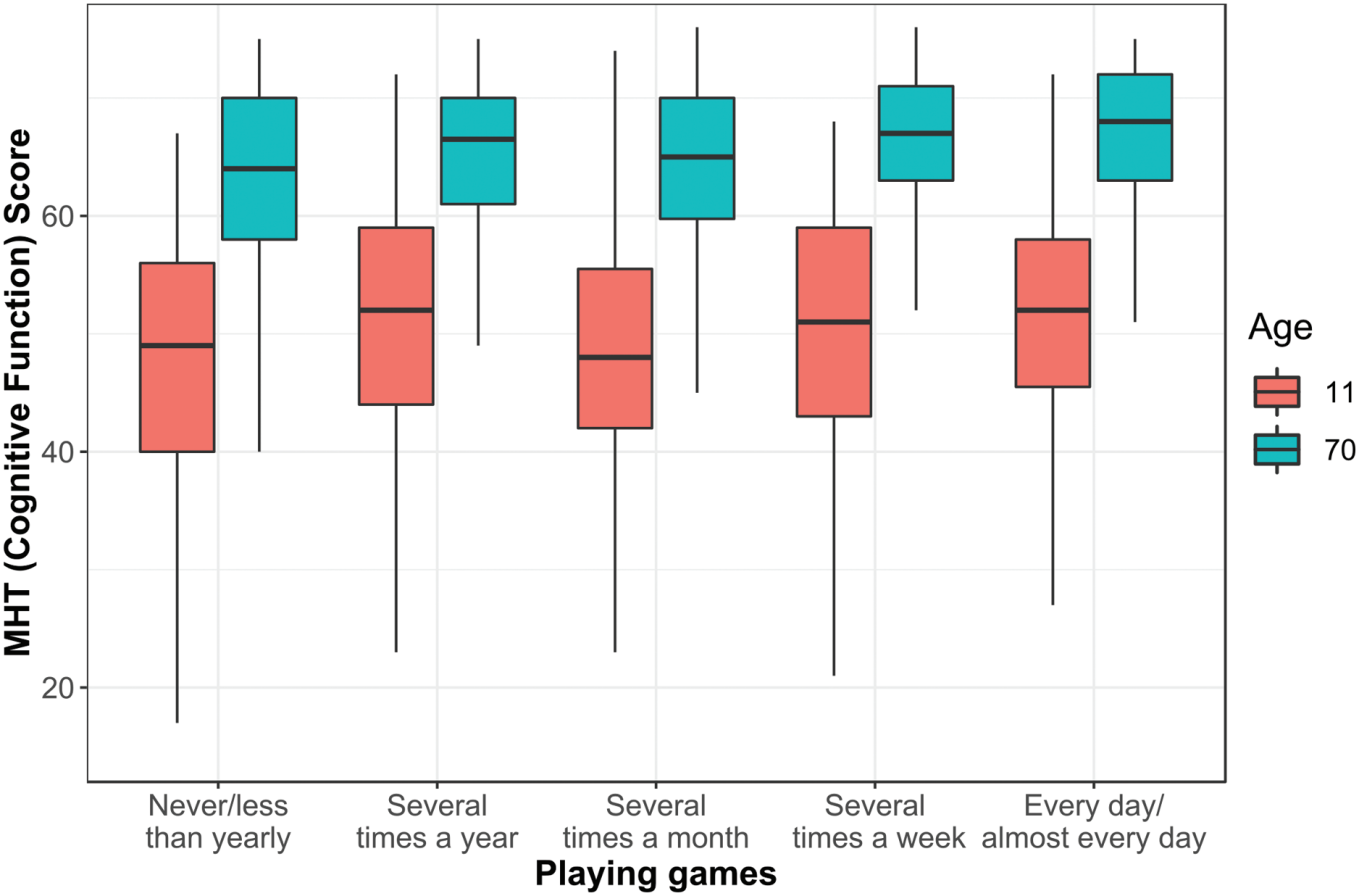
Box-and-whisker plot of Moray House Test (MHT) scores from age 11 and 70, and frequency of playing games. Raw MHT scores represent cognitive function, on a scale from 0 to 76.

Our first hypotheses predicted that playing games would predict higher cognitive function at age 70 and positive change in cognitive function from age 11 to age 70. The differences in cognitive function between age 11 and 70 are shown in Figure 2. Average performance generally increased for individuals from age 11 to 70, but the differences visibly increase between individuals who are less and more frequent games players.

Formal regression modeling demonstrated that playing games was positively associated with age 70 cognitive function (std β = 0.094, *t* = 4.07, *p* < .001; Supplementary Table S1). Higher age 11 cognitive function, being female, having higher social class, and having had more education were all associated with higher age 70 cognitive function (Supplementary Table S1). A key result is that playing games was also associated with positive cognitive change between age 11 and age 70 (std β = 0.095, *t* = 4.07, *p* < .001; Supplementary Table S1). Also associated were lower age 11 function, being female, having higher social class, and having had more education. In both of the above models, the association between playing games and cognitive test score was equivalent to a gain of approximately 1.42 IQ-like points per standard deviation increase in playing games. This would be like increasing one’s frequency of playing games from monthly to several times a week.

Our second hypothesis predicted that, if we control for the possible confounding pathways of age 11 cognitive function, education, and social class, then playing games will still have a positive association with age 70 cognitive function. We modeled the expected life course relationships among these variables, as demonstrated by the path model in Figure 1. Age 11 cognitive function has a positive downstream association with education, social class, and age 70 cognitive function, as well as playing games. Education and social class have their own positive downstream associations, in addition, and social class is slightly associated with games playing, albeit negatively. Thus, despite controlling for the direct and indirect associations of age 11 function, education, and social class with playing games, playing more games was still associated with higher cognitive function at age 70 (std β = 0.083, *z* = 3.24, *p* = .001, Supplementary Table S2). In this model, there was a 1.25 IQ-like point gain from age 11 to age 70 per standard deviation increase in playing games.

### Playing Games and Cognitive Function Change From Age 70 Years to Age 79

Our third set of hypotheses concerned cognitive change across the eighth decade. We predicted that more games playing reported at age 70, and any increases in playing games between age 70 and 76, will predict less relative decline in cognitive functions between age 70 and age 79.

**Figure 3.**
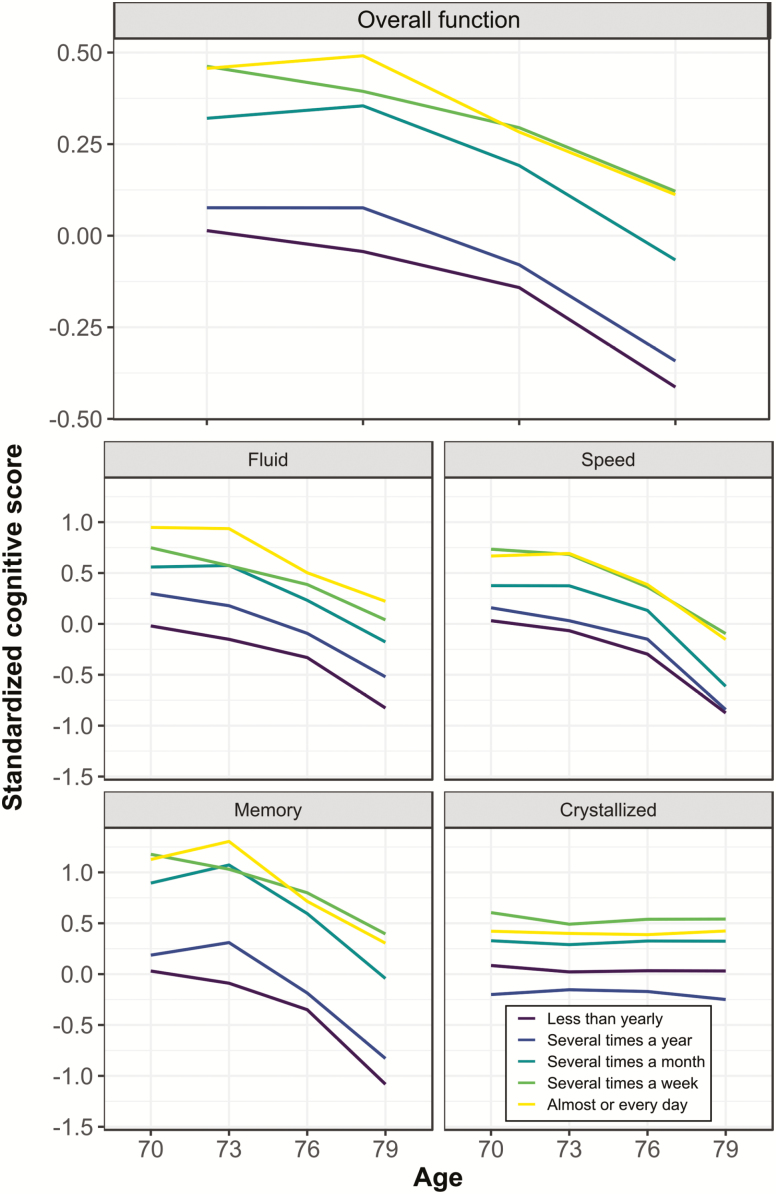
Trajectories of cognitive change across groups with different games playing habits. Data are plotted only for completers, that is, those individuals who participated in all four waves of data collection. General and subdomain cognitive scores are derived from the first wave hierarchical model in our latent variable analyses: tests were standardized according to the characteristics of Wave 1, factor loadings were set by Wave 1, and factor scores for all waves were then estimated. Frequency of games playing increases with brighter lines, that is, dark maroon = “never/less than yearly,” light purple = “several times a year,” turquoise = “several times a month,” light green = “several times a week,” yellow = “every day/almost every day.”

The differences in cognitive decline across the eighth decade, as indicated by playing analog games at age 70, are shown in Figure 3. Across general cognitive function and all cognitive subdomains, intercept differences are apparent; these show that individuals who played more games appear to have higher baseline cognitive functions at age 70. Slope differences among different levels of game playing are notable in general cognitive function and in the memory subdomain. Mean decline occurs among all levels of playing games across the eighth decade, but decline in these variables was more severe among less frequent players.

Latent growth curve models found that playing games was associated with higher general and subdomain cognitive function intercepts (Supplementary Table S4). That is, individuals who played games more often had better baseline cognitive performance from age 70 (β = 0.338, *z* = 5.886, *p* < .001), even controlling for age 11 cognitive function and other sociodemographic and health variables. This reproduces the results derived from the age 70 test scores,-reported above; however, in this analysis, the outcome was a comprehensive, multi-test, multi-domain, hierarchical model of cognitive function.

A key result was that playing more games was associated with less decline in general cognitive function from age 70 to age 79 (β = 0.068, *z* = 2.523, *p* = .012; Table 2). The association between playing games and less cognitive decline was also significant for the memory subdomain (β = 0.204, *z* = 3.114, *p* = .002). For the other cognitive subdomains, all estimates were in the same direction, but not significant. In IQ-score terms, a standard deviation more games playing is associated with 1.02 points less reduction in general ability, and 3.06 points less reduction in memory ability, over the eighth decade.

**Table 2.** Regression Coefficients and Standard Errors for Paths in Growth Curve Model of Cognitive Change

		Subdomain slopes			
Variable	General cognitive function slope	Fluid	Speed	Memory	Crystallized
Playing games	0.068 (0.027)*	0.052 (0.040)	0.034 (0.037)	0.204 (0.065)**	0.029 (0.015)
Age 11 cognitive function	−0.027 (0.026)	−0.102 (0.057)	−0.011 (0.043)	−0.045 (0.064)	−0.000 (0.012)
Education	−0.035 (0.027)	−0.072 (0.057)	−0.012 (0.043)	0.031 (0.068)	−0.028 (0.018)
Social class	−0.023 (0.026)	0.008 (0.036)	−0.056 (0.048)	−0.056 (0.076)	−0.001 (0.013)
Activity score	0.001 (0.026)	0.029 (0.036)	0.027 (0.047)	−0.037 (0.074)	−0.002 (0.012)
Sex	0.034 (0.024)	0.018 (0.048)	0.052 (0.038)	0.134 (0.068)*	0.007 (0.012)
Hypertension	0.003 (0.022)	−0.007 (0.039)	−0.014 (0.041)	0.088 (0.064)	−0.004 (0.011)
Cardiovascular disease	−0.025 (0.029)	−0.040 (0.050)	−0.034 (0.051)	0.015 (0.077)	−0.016 (0.016)
Diabetes	−0.101 (0.047)*	−0.039 (0.080)	−0.128 (0.081)	−0.266 (0.134)*	−0.038 (0.030)
Stroke	0.090 (0.068)	0.056 (0.141)	0.232 (0.118)*	0.223 (0.177)	0.006 (0.031)
CFI	0.941				
RMSEA	0.032				
SRMR	0.060				

*Notes*: Each regression estimate is the effect of 1SD change in the predictor variable on standardized cognitive function change over the course of a decade. Standard errors are in parentheses. The regression effects on general cognitive change and subdomain change models had to be estimated in separate models due to correlations within the hierarchical model. CFI = comparative fit index, RMSEA = root mean square error of approximation, SRMS = standardized root mean square residual.

*p < .05, **p < .01.

Increasing playing games between age 70 and 76 was associated with reduced decline in the processing speed subdomain (β = 0.110, *z* = 2.689, *p* = .007; Supplementary Table S5). This result was only significant for the ordinally constructed variable that captured relative behavior change. The binary variable, which only identified if an individual played more games or not, likely lost too much information in the transformation process and did not have the power to detect this effect, though the binary variable always trended in the expected direction (Supplementary Table S6).

### Sensitivity Analyses in Growth Curve Models

We carried out non preregistered sensitivity analyses that included all individuals in our growth curve models, that is, including the 37 who were removed for having low MMSE scores that indicated cognitive impairment. Including these individuals generally did not alter our primary findings: Playing games was still associated with less general cognitive function decline (β = 0.057, std error = 0.022) and increasing playing games was associated with less speed decline (β = 0.095, std error = 0.052). Effect sizes were generally the same albeit smaller, though the association between playing games and memory slope was much reduced (β = 0.017, std error = 0.007). This is not surprising as the memory subdomain is particularly linked to mild cognitive impairment (Allaire, Gamaldo, Ayotte, Sims, & Whitfield, 2009); including impaired individuals appears to attenuate our models’ ability to determine reliable estimates of associations with cognitive domains.

## Discussion

In this study, we found consistent evidence that playing more analog games is associated with significantly less relative cognitive decline from age 11 to age 70, and also less cognitive decline from age 70 to age 79. In our models we introduced a consistent set of well-validated sociodemographic and health variables as potential confounders. Whereas there were some confounding influences from these variables, the association between cognitive variables and playing games was robust to the inclusion of all covariates.

Our results revealed that there were particularly strong positive relationships with general cognitive function and with memory. Those LBC1936 participants who increased their games playing frequency from age 70 to age 76 appeared to experience positive associated benefits, but only in the speed subdomain. This is likely due to the greater sensitivity of speed to age-related decline (Ritchie et al., 2016; Verhaeghen & Salthouse, 1997).

The association between playing games and cognitive function cannot be entirely explained by cognitive reserve (Scarmeas & Stern, 2003); that is, if we account for the associations with hallmarks of cognitive reserve: early life cognitive function, education, activity, and social class, there is still a distinct association between playing more games and experiencing less cognitive decline. After controlling for these particular confounders, 64% (Supplementary Table S3) of the relationship between playing games and later life function appears to be due to playing the games themselves. This supports the use-it-or-lose-it hypothesis: in this sample, mental exercise in the form of playing games might help slow cognitive decline and even increase cognitive function.

A strength of the present study is the longitudinal sample used. LBC1936 provided data on many life course factors including a validated measure of early life, premorbid cognitive function. The same cognitive test was given at ages 11 and 70, allowing for direct comparison of performance across nearly 60 years. From age 70 and beyond, LBC1936 has data from 14 validated cognitive tests that fit a four subdomain hierarchical model of cognitive function. The general factor and subdomains were all strongly associated with early life cognitive function, and by accounting for this and other variables’ influence we could examine and identify the particular subdomains of cognitive function that were associated with playing games.

An additional strength of our study is that all of our analyses were preregistered. Variables, model structures, fit and significance cutoffs were all specified in advance. Moreover, sociodemographic variables, particularly educational attainment and social class, made it possible for us to disentangle the association of playing games with later life intelligence, without confounding from these early life influences. We also had access to health and activity variables that allowed us to account for their potential relationships with lifestyle choices and cognitive function.

This study had several limitations as well. For instance, our sociodemographic data were retrospective, and sociodemographic, activities, and health data were self-reported. Additionally, retention across waves has consistently been approximately 80% (Taylor et al., 2018), and between waves the major causes of attrition, death and frailty, are insurmountable. Whereas we were able to use the larger Wave 1, age 70, sample for our univariate analyses of change between age 11 and 70, our sample size and power was hindered by a decrease in sample size across the waves. In particular, our analyses of increasing one’s games-playing frequency may have suffered from insufficient power, as those analyses were limited by the reduced number of responses from Wave 3, at age 76, and the relatively small number of individuals who actually increased their frequency of playing games (*n* = 160).

The recruitment process for samples of older people tends to self-select volunteers who are often better educated and aging well, which is true of LBC1936 (Taylor et al., 2018). Findings might be biased toward affluent and/or higher cognitive function individuals, who might be inclined to play games more often. Previous studies have also focused on particular games, for example, Sudoku (Ferreira et al., 2015) or board games (Dartigues et al., 2013). Our study was not so detailed, we could only examine playing games in aggregate, limiting the specificity of our conclusions. Previous research has also suggested that the social component to playing games may play an important role in the positive benefits of games (Kuo et al., 2018; Ngandu et al., 2015). From previous work (Gow et al., 2012) (and reproduced in our analyses), more social activity does not appear to be associated with reduced cognitive decline.

Unlike many digital cognitive training studies (Anguera et al., 2013; Ball et al., 2002; Ngandu et al., 2015) and one analog games study (Kuo et al., 2018), this was not a randomized controlled trial. Without an experimental manipulation, we cannot conclude that we have controlled for all confounds that might bias our results, and causal effects cannot be inferred. Randomized controlled trials are extremely difficult to carry out when the entire life course is under study, but we were able to test whether multiple early life factors, including cognitive function from age 11, confound associations between playing games and later life cognitive function. This finding is in line with those of Staff et al. (2018), who studied the cognitive associations with a self-reported intellectual engagement trait whilst controlling for early life cognitive function. However, intellectual engagement and social activities are confounded by early life variables (Gow et al., 2012; Von Stumm & Deary, 2012). Our analyses controlled for these activities along with the same early life variables, and our results nevertheless suggest that playing games, a self-reported behavior, not an intellectual or personality trait, is associated with higher cognitive function and less decline.

This study suggests that the capacities used while playing games generalize to the cognitive tests we used to assess cognitive function. The effects also have long-term consequences, as individuals who reported playing more games at age 70 performed better on cognitive tasks 3, 6, and 9 years later, and among individuals who changed their game playing habits, speed declines were not as large for those who increased their games playing.

Playing more games is associated with less cognitive decline overall, though particularly in general cognitive function and in the memory subdomain. This finding links previous research between games, intellectual activity, and dementia prevention, as deficiencies in the memory subdomain are related to mild cognitive impairment (Allaire et al., 2009) and subsequent dementia (DeCarli et al., 2004). Whereas the memory subdomain was driving much of the overall relationship between playing games and cognitive change, the other subdomains all also trended in the same direction, though they were not significantly associated themselves. Future work ought to study individual games and their associations with cognitive functions.

Apart from well documented factors like smoking, physical exercise, and fitness (Corley et al., 2018) there are few known behavioral decisions people can make that will positively influence their cognitive functions. Previous studies on games (Dartigues et al., 2013; Kuo et al., 2018) and other intellectual activities (Staff et al., 2018) have not provided compelling evidence that these activities protect against subsequent cognitive decline. This study goes further by specifically suggesting that playing games is related to reduced decline.

It will be a challenge to determine how best to apply these findings in practice, as our results suggest that a lifetime of playing games is the best way to capture the benefits. At present, evidence is still limited, but there do not appear to be any harmful effects of playing games. Thus, analog games are an affordable and fun activity that could protect against cognitive decline.

In conclusion, this preregistered study of cognitive change from age 11 to age 70, and then age 70–79 has shown that playing more games might improve the long-term outlook for one’s cognitive health. Additional longitudinal investigations, including randomized controlled trials, with larger numbers are needed to clarify the avenues through which analog games might protect against cognitive decline.

## Supplementary Material

gbz149_suppl_Supplementary_Material

## Data and Material Availability

Data and analytic code used in this publication are available to bona fide researchers upon request to the Lothian Birth Cohorts study coordinator via a standard application procedure.
