# Supplementary material for: Playing Analog Games Is Associated With Reduced Declines in Cognitive Function: A 68-Year Longitudinal Cohort Study
Source: J Gerontol B Psychol Sci Soc Sci. 2019 Nov 18;75(3):474–82. doi: 10.1093/geronb/gbz149 (PMC7021446; doi:10.1093/geronb/gbz149)
Supplement: gbz149_suppl_Supplementary_Material [file gbz149_suppl_supplementary_material.docx]

**Playing analog games is associated with reduced declines in cognitive function: a 68 year longitudinal cohort study**

Supplementary Materials

Appendix 1. Cognitive battery used in older age.

Appendix 2. Additional control variables and analyses.

Appendix 3. Model controlling for confounding effects on age 70 cognitive function.

Appendix 4. Item reliability of playing games behaviours.

Figure S1. Representative path diagram of latent growth curve analyses of cognitive function, playing games, and other covariates.

Figure S2. Confounding control model of associations among playing games, confounders, and age 70 cognitive function

Figure S3. Box-and-whisker plot of Moray House Test (MHT) scores from age 11, covariate adjusted scores for age 70, and frequency of playing games.

Table S1. Regression models of playing games and covariates' associations with change in cognitive function

Table S2. Life course model of playing games and relationships between age 11 and 70 cognitive function and other sociodemographic factors.

Table S3. Confounding control model of playing games, cognitive function, and sociodemographic and health covariates.

Table S4. Latent growth curve models of general and subdomain cognitive functions, playing games, and sociodemographic and health covariates.

Table S5. Latent growth curve models of general and subdomain cognitive functions, playing games and difference in playing games between age 70 and 76, and sociodemographic and health covariates.

Table S6. Latent growth curve models of general and subdomain cognitive functions, playing games and increases in playing games, and sociodemographic and health covariates.

Table S7. Principal components analysis of socio-intellectual activities.

**Appendix 1. Cognitive battery used in older age.**

In older age, cognitive function is known to show decline across multiple, but not all, subdomains (Verhaeghen & Salthouse, 1997). We assessed fluid cognitive ability, processing speed, and memory, all of which decline on average with age. We also assessed crystallised ability, which remains relatively stable in later life (Horn & Cattell, 1967). There are strong correlations among the subdomains, and because of this, cognitive function can be modelled hierarchically, with a general cognitive function factor that captures overall ability. Beneath that general factor, specific subdomains capture variation beyond general cognitive function (Ritchie et al., 2016). The relationships among cognitive tests and subdomains are described in the Statistical Analyses section in the main text. Cognitive functions in waves 1–4 were assessed using 14 individually-administered cognitive tests at the same clinical research facility and using the same equipment and procedure for all four waves. The tests are fully described and referenced in an open-access protocol article (Taylor, Pattie, & Deary, 2018). The fluid subdomain consisted of matrix reasoning and block design from the Wechsler Adult Intelligence Scale (WAIS)(Wechsler, 1997), and spatial span forward and backward from the Wechsler Memory Scale (WMS)(Wechsler, 1945), Processing speed was measured through symbol search and digit symbol substitution from the WAIS, plus four-choice reaction time (Ritchie et al., 2016) and inspection time (Deary, Johnson, & Starr, 2010). Memory was assessed using verbal paired associates and logical memory from the WMS(Wechsler, 1945), and the letter–number sequencing and digit span backward subtests of the WAIS(Wechsler, 1997). Crystallised ability was measured through the National Adult Reading Test, Wechsler Test of Adult Reading, and a phonemic verbal fluency test (Ritchie et al., 2016).

**Appendix 2. Additional social and intellectual activities analyses.**

Education was recorded as the total number of years spent in formal education. Social class was derived from a participant’s most prestigious job before retirement, on a 6-point ordinal scale ranging from unskilled to professional work (Elias, McKnight, & Kinshott, 1999).

We conducted a principal components analysis on 13 activity variables: how often the participant “visits libraries”, “reads newspaper/magazine”, “reads a book”, “writes for pleasure”, “listens to the radio”, “visits friends or family”, “studies course at work or evening classes”, “goes to the cinema or restaurants”, “goes to sporting events or concerts”, “goes on trips to theatre, galleries or museums”, “participates in social groups”, and “participates in church or religious activities”. All were rated on a five-point scale. There was also a measure of overall physical activity, rated on a six-point scale. The playing games variable in the questionnaire was not included in this analysis to avoid confounding overlap. We performed principal components analysis (Table S7) on the activity variables and found a strong first component that accounted for 21% of the variance. The loadings on this component ranged from 0.07 to 0.69, with a mean loading of 0.30. The lowest loading, “reads newspaper/magazine” was much lower than the next lowest (0.29 for “overall physical activity”), and simply reflects that whether individuals read newspapers or magazines has little to do with their social or intellectual activities (in contrast with reading books, which loaded at 0.42). We took individual scores on the first component as a measure of overall activity for a given participant.

**Appendix 3. Model controlling for confounding effects on age 70 cognitive function.**

We wished to evaluate to what extent there was a direct relationship between playing games and having higher cognitive function at age 70, thus we formulated a model that allowed us to estimate the association between playing games and cognitive function that was both direct (i.e., change in cognitive function due to change in the playing games variable itself), and indirect (i.e., change in cognitive function that could be attributed to change in a confounder). This model is statistically equivalent to a mediation model, but this analysis does not imply any causal mediation. Figure S2 illustrates the pathways of direct and indirect effects; at this stage, the numbers in the Figure may be ignored.

This model was intended to provide a conservative estimate of the association between playing games and age 70 cognitive function, adjusting for life-history and health variables. Age 11 cognitive function, activity score, social class, and education were all specified as confounders, and sex and all health condition histories were used as control variables. Figure S2 shows the path diagram and path coefficients (see also Table S3). Age 11 cognitive function and activity score were both significant confounders of playing games, but only age 11 cognitive function significantly confounded playing games’ association with age 70 cognitive function. 64% of the relationship between playing games and age 70 cognitive function (direct effect: std β = 0.098, t = 4.38, p < 0.001; total effect: std β = 0.152, t = 5.08, p < 0.001) was not due to external factors including age 11 cognitive function, sociodemographic variables, or health conditions. This path model indicated that a standard deviation increase in playing games was associated with a 1.47 IQ-like point gain from age 11 to age 70.

**Appendix 4. Item reliability of playing games behaviours.**

Single-item measures can be reliable, particularly if they are assessing quantitative facts, like how often one plays games, rather than psychological constructs, e.g. personality or job satisfaction (Wanous, Reichers, & Hudy, 1997). Moreover, even where there is some psychologically influenced subjectivity to a question, a single-item can still be reliable (Ginns & Barrie, 2004). We thus followed recommendations on testing single-item reliability (Ginns & Barrie, 2004) in several ways using our age 70 and 76 data on playing games. First, we found a Spearman correlation of *ρ* = 0.63. Second we calculated the proportion of variance explained (equivalent to the communality) of both observations by a single principal axis factor: 64%. These measures indicate good test-retest reliability, especially considering that this is a single item. Third, we calculated the intraclass correlation *ICC(3,1)* = 0.64, indicating good consistency in individuals’ ratings of their games playing frequencies. All measures were similar due to good overall reliability and a lack of exploitation of any particular reliability formula (Ginns & Barrie, 2004). This is consistent with previous work that has suggested that individuals’ self-reporting of playing games is accurate (Waris et al., 2019). Moreover, we ought not to expect reliability to be higher, due to actual changes in games playing behaviour (again see Table 1).

**Figure S1. Representative path diagram of latent growth curve analyses of cognitive function, playing games, and other covariates.** Circles indicate latent variables, and squares indicate measured variables. Dashed single-arrowed lines represent regression relationships, solid single-arrowed lines represent correlations, and solid double-arrowed lines represent loadings from latent variables higher in the model hierarchy. For simplicity, this model is a scaled down version of the full hierarchical model, which contains 4 subdomains, not only 2 as shown here.


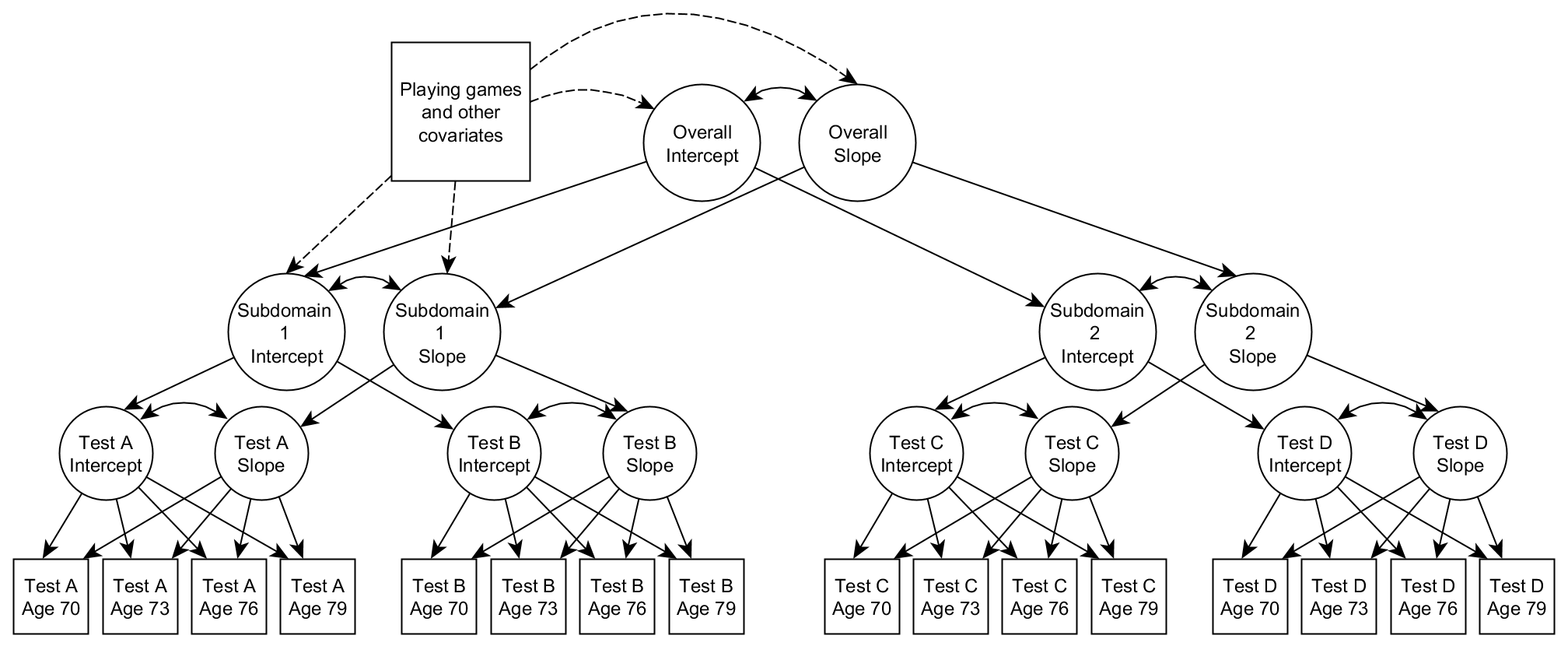


**Figure S2. Confounding control model of associations among playing games, confounders, and age 70 cognitive function.** The centre column of variables holds all the confounders. Numbers accompanying path lines indicate std β weights and std errors (in parentheses). All paths are significant at p < 0.05 except those printed in italics. Sex and any histories of hypertension, cardiovascular disease, diabetes or stroke were all included as control variables.


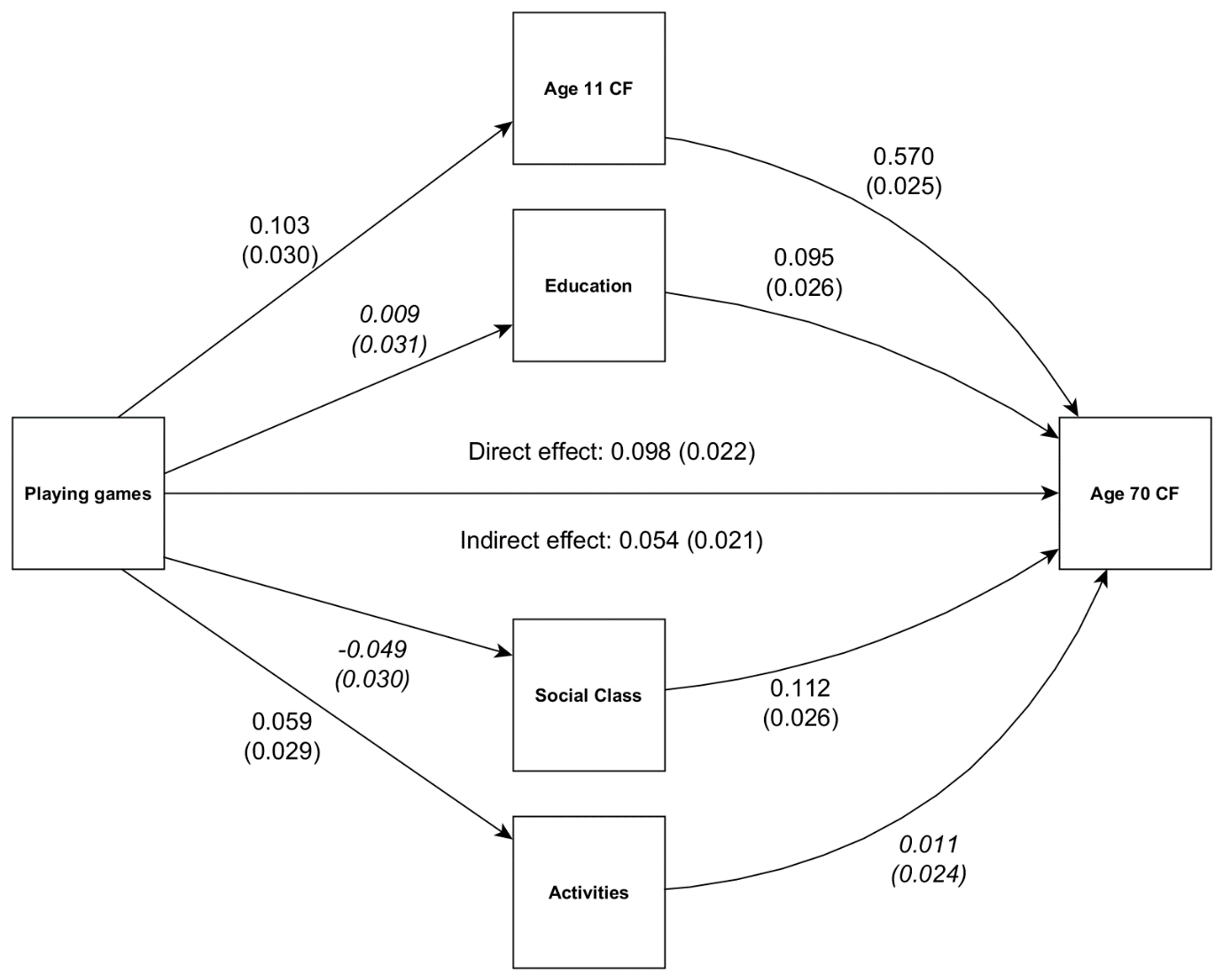


**Figure S3. Box-and-whisker plot of Moray House Test (MHT) scores from age 11, covariate adjusted scores for age 70, and frequency of playing games.** Raw age 11 MHT scores represent cognitive function, on a scale from 0 to 76. Adjusted age 70 values are non-standardized residual scores for the age 70 MHT scores, regressed on education, social class, activity score, sex, hypertension, CVD, diabetes, and stroke history.


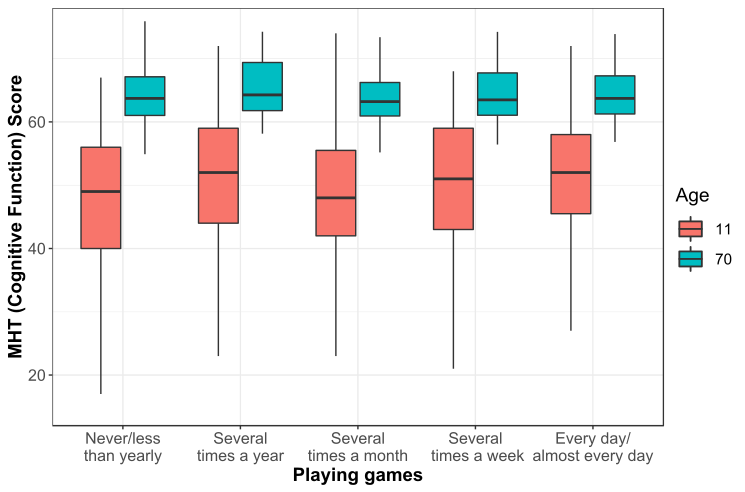


|  | | | Table S1. Regression models of playing games and covariates' associations with change in cognitive function | |
| --- | --- | --- | --- | --- |
|  |  |  | |  |
|  |  | Age 70 cognitive function | | Cognitive function change |
| Variable |  | Std β (Std Error) | | Std β (Std Error) |
| Playing games |  | 0.094 (0.023) ** | | 0.095 (0.023) ** |
| Age 11 cognitive function |  | 0.561 (0.027) ** | | -0.786 (0.027) ** |
| Education |  | 0.080 (0.027) * | | 0.080 (0.028) * |
| Social class |  | 0.112 (0.027) ** | | 0.113 (0.027) ** |
| Activity score |  | -0.003 (0.023) | | -0.003 (0.024) |
| Sex (women) |  | -0.096 (0.023) ** | | -0.097 (0.024) ** |
| Hypertension |  | -0.027 (0.024) | | -0.027 (0.024) |
| Cardiovascular disease |  | -0.017 (0.028) | | -0.018 (0.027) |
| Diabetes |  | -0.058 (0.043) | | -0.059 (0.044) |
| Stroke |  | -0.031 (0.058) | | -0.032 (0.058) |
| Adjusted R² |  | 0.484 | | 0.514 |
| * = p < 0.01, ** = p < 0.001 |  |  | |  |
|  |  |  | |  |

| Table S2. Life course model of playing games and relationships between age 11 and 70 cognitive function and other sociodemographic factors. | | | | | |
| --- | --- | --- | --- | --- | --- |
|  |  |  |  |  |  |
| Path | | | Estimate | Std Error | p |
| Age 70 CF | ~ | Playing games | 0.083 | 0.023 | 0.000 |
| Age 70 CF | ~ | Social class | 0.097 | 0.026 | 0.000 |
| Age 70 CF | ~ | Education | 0.090 | 0.027 | 0.001 |
| Age 70 CF | ~ | Age 11 CF | 0.562 | 0.027 | 0.000 |
| Playing games | ~ | Social class | -0.075 | 0.038 | 0.050 |
| Playing games | ~ | Education | -0.018 | 0.040 | 0.646 |
| Playing games | ~ | Age 11 CF | 0.159 | 0.039 | 0.000 |
| Social class | ~ | Education | 0.369 | 0.032 | 0.000 |
| Social class | ~ | Age 11 CF | 0.222 | 0.033 | 0.000 |
| Education | ~ | Age 11 CF | 0.438 | 0.031 | 0.000 |
| Age 70 CF | ~~ | Age 70 CF | 0.114 | 0.005 | 0.000 |
| Playing games | ~~ | Playing games | 0.241 | 0.011 | 0.000 |
| Social class | ~~ | Social class | 0.185 | 0.009 | 0.000 |
| Education | ~~ | Education | 0.200 | 0.009 | 0.000 |
| Age 11 CF | ~~ | Age 11 CF | 0.232 | 0.000 |  |
|  |  |  |  |  |  |

| Table S3. Confounding control model of playing games, cognitive function, and sociodemographic and health covariates. | | | | | |
| --- | --- | --- | --- | --- | --- |
|  |  |  |  |  |  |
|  | Estimate | Std Error | t | df | p |
| Total effect estimates (c) | Age 70 CF |  |  |  |  |
| Playing games | 0.152 | 0.030 | 5.075 | 1080 | < 0.0001 |
| Direct effect estimates (c') | Age 70 CF |  |  |  |  |
| Playing games | 0.098 | 0.022 | 4.388 | 1080 | < 0.0001 |
| Age 11 CF | 0.570 | 0.025 | 22.407 | 1080 | < 0.0001 |
| Education | 0.095 | 0.026 | 3.642 | 1080 | 0.0003 |
| Social class | 0.112 | 0.026 | 4.318 | 1080 | < 0.0001 |
| Activity score | 0.011 | 0.024 | 0.462 | 1080 | 0.644 |
| "a" effect estimates | Playing games |  |  |  |  |
| Age 11 CF | 0.103 | 0.030 | 3.405 | 1084 | 0.0006 |
| Education | 0.009 | 0.031 | 0.307 | 1084 | 0.759 |
| Social class | -0.049 | 0.030 | -1.620 | 1084 | 0.106 |
| Activity score | 0.059 | 0.030 | 1.997 | 1084 | 0.046 |
| "b" effect estimates | Age 70 CF |  |  |  |  |
| Age 11 CF | 0.570 | 0.025 | 22.407 | 1080 | < 0.0001 |
| Education | 0.095 | 0.026 | 3.642 | 1080 | 0.0003 |
| Social class | 0.112 | 0.026 | 4.318 | 1080 | < 0.0001 |
| Activity score | 0.011 | 0.024 | 0.462 | 1080 | 0.644 |
|  |  |  |  |  |  |
| "ab" effect estimates | Estimate | SD |  | 95% | CI |
| Playing games; Age 70 CF | 0.055 | 0.021 |  | [0.010, | 0.092] |
| "ab" effect estimates for each confounder |  |  |  |  |  |
| Playing games; Age 11 CF | 0.059 | 0.018 |  | [0.023, | 0.091] |
| Playing games; Education | 0.001 | 0.003 |  | [-0.006, | 0.007] |
| Playing games; Social class | -0.006 | 0.004 |  | [-0.015, | 0.001] |
| Playing games; Activity score | 0.001 | 0.002 |  | [-0.003, | 0.005] |
| R² | 0.474 |  |  |  |  |

| Table S4. Latent growth curve models of general and subdomain cognitive functions, playing games, and sociodemographic and health covariates. | | | | | |
| --- | --- | --- | --- | --- | --- |
|  |  |  |  |  |  |
| Path | | | Estimate | Std Error | p |
| General factor associations | |  |  |  |  |
| General CF slope | ~ | Sex | 0.034 | 0.024 | 0.157 |
| General CF slope | ~ | Age 11 CF | -0.027 | 0.026 | 0.295 |
| General CF slope | ~ | Playing games | 0.068 | 0.027 | 0.012 |
| General CF slope | ~ | Education | -0.035 | 0.027 | 0.192 |
| General CF slope | ~ | Social class | -0.023 | 0.026 | 0.368 |
| General CF slope | ~ | Activity score | 0.001 | 0.026 | 0.966 |
| General CF slope | ~ | Hypertension | 0.003 | 0.022 | 0.905 |
| General CF slope | ~ | Cardiovascular disease | -0.025 | 0.029 | 0.400 |
| General CF slope | ~ | Diabetes | -0.101 | 0.047 | 0.034 |
| General CF slope | ~ | Stroke | 0.090 | 0.068 | 0.186 |
| General CF intercept | ~ | Sex | -0.237 | 0.063 | 0.000 |
| General CF intercept | ~ | Age 11 CF | 1.397 | 0.097 | 0.000 |
| General CF intercept | ~ | Playing games | 0.338 | 0.057 | 0.000 |
| General CF intercept | ~ | Education | 0.421 | 0.071 | 0.000 |
| General CF intercept | ~ | Social class | 0.330 | 0.067 | 0.000 |
| General CF intercept | ~ | Activity score | 0.100 | 0.061 | 0.099 |
| General CF intercept | ~ | Hypertension | -0.156 | 0.053 | 0.003 |
| General CF intercept | ~ | Cardiovascular disease | -0.067 | 0.065 | 0.302 |
| General CF intercept | ~ | Diabetes | -0.204 | 0.115 | 0.075 |
| General CF intercept | ~ | Stroke | -0.125 | 0.150 | 0.407 |
| Sex | ~~ | Sex | 0.250 | 0.000 |  |
| Sex | ~~ | Age 11 CF | 0.012 | 0.000 |  |
| Sex | ~~ | Playing games | 0.028 | 0.000 |  |
| Sex | ~~ | Education | -0.007 | 0.000 |  |
| Sex | ~~ | Social class | 0.029 | 0.000 |  |
| Sex | ~~ | Activity score | 0.051 | 0.000 |  |
| Sex | ~~ | Hypertension | -0.007 | 0.000 |  |
| Sex | ~~ | Cardiovascular disease | -0.022 | 0.000 |  |
| Sex | ~~ | Diabetes | -0.008 | 0.000 |  |
| Sex | ~~ | Stroke | 0.000 | 0.000 |  |
| Age 11 CF | ~~ | Age 11 CF | 0.237 | 0.000 |  |
| Age 11 CF | ~~ | Playing games | 0.034 | 0.000 |  |
| Age 11 CF | ~~ | Education | 0.100 | 0.000 |  |
| Age 11 CF | ~~ | Social class | 0.089 | 0.000 |  |
| Age 11 CF | ~~ | Activity score | 0.076 | 0.000 |  |
| Age 11 CF | ~~ | Hypertension | -0.009 | 0.000 |  |
| Age 11 CF | ~~ | Cardiovascular disease | -0.017 | 0.000 |  |
| Age 11 CF | ~~ | Diabetes | -0.015 | 0.000 |  |
| Age 11 CF | ~~ | Stroke | -0.001 | 0.000 |  |
| Playing games | ~~ | Playing games | 0.248 | 0.000 |  |
| Playing games | ~~ | Education | 0.006 | 0.000 |  |
| Playing games | ~~ | Social class | -0.005 | 0.000 |  |
| Playing games | ~~ | Activity score | 0.017 | 0.000 |  |
| Playing games | ~~ | Hypertension | 0.002 | 0.000 |  |
| Playing games | ~~ | Cardiovascular disease | -0.004 | 0.000 |  |
| Playing games | ~~ | Diabetes | 0.001 | 0.000 |  |
| Playing games | ~~ | Stroke | 0.007 | 0.000 |  |
| Education | ~~ | Education | 0.245 | 0.000 |  |
| Education | ~~ | Social class | 0.111 | 0.000 |  |
| Education | ~~ | Activity score | 0.074 | 0.000 |  |
| Education | ~~ | Hypertension | -0.011 | 0.000 |  |
| Education | ~~ | Cardiovascular disease | -0.004 | 0.000 |  |
| Education | ~~ | Diabetes | -0.006 | 0.000 |  |
| Education | ~~ | Stroke | 0.001 | 0.000 |  |
| Social class | ~~ | Social class | 0.246 | 0.000 |  |
| Social class | ~~ | Activity score | 0.082 | 0.000 |  |
| Social class | ~~ | Hypertension | -0.011 | 0.000 |  |
| Social class | ~~ | Cardiovascular disease | -0.003 | 0.000 |  |
| Social class | ~~ | Diabetes | -0.007 | 0.000 |  |
| Social class | ~~ | Stroke | -0.004 | 0.000 |  |
| Activity score | ~~ | Activity score | 0.281 | 0.000 |  |
| Activity score | ~~ | Hypertension | -0.028 | 0.000 |  |
| Activity score | ~~ | Cardiovascular disease | -0.026 | 0.000 |  |
| Activity score | ~~ | Diabetes | -0.021 | 0.000 |  |
| Activity score | ~~ | Stroke | -0.008 | 0.000 |  |
| Hypertension | ~~ | Hypertension | 0.239 | 0.000 |  |
| Hypertension | ~~ | Cardiovascular disease | 0.044 | 0.000 |  |
| Hypertension | ~~ | Diabetes | 0.028 | 0.000 |  |
| Hypertension | ~~ | Stroke | 0.006 | 0.000 |  |
| Cardiovascular disease | ~~ | Cardiovascular disease | 0.177 | 0.000 |  |
| Cardiovascular disease | ~~ | Diabetes | 0.015 | 0.000 |  |
| Cardiovascular disease | ~~ | Stroke | 0.003 | 0.000 |  |
| Diabetes | ~~ | Diabetes | 0.073 | 0.000 |  |
| Diabetes | ~~ | Stroke | 0.006 | 0.000 |  |
| Stroke | ~~ | Stroke | 0.038 | 0.000 |  |
| Subdomain associations |  |  |  |  |  |
| Fluid CF slope | ~ | Sex | 0.018 | 0.048 | 0.709 |
| Fluid CF slope | ~ | Age 11 CF | -0.102 | 0.057 | 0.071 |
| Fluid CF slope | ~ | Playing games | 0.052 | 0.040 | 0.189 |
| Fluid CF slope | ~ | Education | -0.072 | 0.057 | 0.209 |
| Fluid CF slope | ~ | Social class | 0.008 | 0.044 | 0.849 |
| Fluid CF slope | ~ | Activity score | 0.029 | 0.036 | 0.430 |
| Fluid CF slope | ~ | Hypertension | -0.007 | 0.039 | 0.850 |
| Fluid CF slope | ~ | Cardiovascular disease | -0.040 | 0.050 | 0.430 |
| Fluid CF slope | ~ | Diabetes | -0.039 | 0.080 | 0.621 |
| Fluid CF slope | ~ | Stroke | 0.056 | 0.141 | 0.689 |
| Speed CF slope | ~ | Sex | 0.052 | 0.038 | 0.170 |
| Speed CF slope | ~ | Age 11 CF | -0.011 | 0.043 | 0.798 |
| Speed CF slope | ~ | Playing games | 0.034 | 0.037 | 0.358 |
| Speed CF slope | ~ | Education | -0.012 | 0.043 | 0.786 |
| Speed CF slope | ~ | Social class | -0.056 | 0.048 | 0.238 |
| Speed CF slope | ~ | Activity score | 0.027 | 0.047 | 0.572 |
| Speed CF slope | ~ | Hypertension | -0.014 | 0.041 | 0.728 |
| Speed CF slope | ~ | Cardiovascular disease | -0.034 | 0.051 | 0.510 |
| Speed CF slope | ~ | Diabetes | -0.128 | 0.081 | 0.113 |
| Speed CF slope | ~ | Stroke | 0.232 | 0.118 | 0.049 |
| Memory CF slope | ~ | Sex | 0.134 | 0.068 | 0.050 |
| Memory CF slope | ~ | Age 11 CF | -0.045 | 0.064 | 0.476 |
| Memory CF slope | ~ | Playing games | 0.204 | 0.065 | 0.002 |
| Memory CF slope | ~ | Education | 0.031 | 0.068 | 0.653 |
| Memory CF slope | ~ | Social class | -0.056 | 0.076 | 0.457 |
| Memory CF slope | ~ | Activity score | -0.037 | 0.074 | 0.619 |
| Memory CF slope | ~ | Hypertension | 0.088 | 0.064 | 0.169 |
| Memory CF slope | ~ | Cardiovascular disease | 0.015 | 0.077 | 0.840 |
| Memory CF slope | ~ | Diabetes | -0.266 | 0.134 | 0.048 |
| Memory CF slope | ~ | Stroke | 0.223 | 0.177 | 0.208 |
| Crystallized CF slope | ~ | Sex | 0.007 | 0.012 | 0.522 |
| Crystallized CF slope | ~ | Age 11 CF | 0.000 | 0.012 | 0.986 |
| Crystallized CF slope | ~ | Playing games | 0.029 | 0.015 | 0.051 |
| Crystallized CF slope | ~ | Education | -0.028 | 0.018 | 0.119 |
| Crystallized CF slope | ~ | Social class | -0.001 | 0.013 | 0.938 |
| Crystallized CF slope | ~ | Activity score | -0.002 | 0.012 | 0.871 |
| Crystallized CF slope | ~ | Hypertension | -0.004 | 0.011 | 0.731 |
| Crystallized CF slope | ~ | Cardiovascular disease | -0.016 | 0.016 | 0.333 |
| Crystallized CF slope | ~ | Diabetes | -0.038 | 0.030 | 0.206 |
| Crystallized CF slope | ~ | Stroke | 0.006 | 0.031 | 0.846 |
| Fluid CF intercept | ~ | Sex | -0.822 | 0.099 | 0.000 |
| Fluid CF intercept | ~ | Age 11 CF | 1.355 | 0.138 | 0.000 |
| Fluid CF intercept | ~ | Playing games | 0.343 | 0.099 | 0.001 |
| Fluid CF intercept | ~ | Education | 0.409 | 0.126 | 0.001 |
| Fluid CF intercept | ~ | Social class | 0.342 | 0.108 | 0.002 |
| Fluid CF intercept | ~ | Activity score | -0.021 | 0.092 | 0.821 |
| Fluid CF intercept | ~ | Hypertension | -0.117 | 0.096 | 0.226 |
| Fluid CF intercept | ~ | Cardiovascular disease | -0.130 | 0.110 | 0.239 |
| Fluid CF intercept | ~ | Diabetes | -0.245 | 0.183 | 0.179 |
| Fluid CF intercept | ~ | Stroke | -0.311 | 0.245 | 0.204 |
| Speed CF intercept | ~ | Sex | -0.175 | 0.102 | 0.087 |
| Speed CF intercept | ~ | Age 11 CF | 1.164 | 0.125 | 0.000 |
| Speed CF intercept | ~ | Playing games | 0.561 | 0.099 | 0.000 |
| Speed CF intercept | ~ | Education | 0.152 | 0.122 | 0.215 |
| Speed CF intercept | ~ | Social class | 0.455 | 0.108 | 0.000 |
| Speed CF intercept | ~ | Activity score | 0.118 | 0.101 | 0.240 |
| Speed CF intercept | ~ | Hypertension | -0.129 | 0.098 | 0.187 |
| Speed CF intercept | ~ | Cardiovascular disease | -0.369 | 0.111 | 0.001 |
| Speed CF intercept | ~ | Diabetes | -0.282 | 0.184 | 0.126 |
| Speed CF intercept | ~ | Stroke | -0.693 | 0.285 | 0.015 |
| Memory CF intercept | ~ | Sex | -0.106 | 0.056 | 0.059 |
| Memory CF intercept | ~ | Age 11 CF | 0.908 | 0.121 | 0.000 |
| Memory CF intercept | ~ | Playing games | 0.202 | 0.055 | 0.000 |
| Memory CF intercept | ~ | Education | 0.127 | 0.069 | 0.067 |
| Memory CF intercept | ~ | Social class | 0.132 | 0.067 | 0.048 |
| Memory CF intercept | ~ | Activity score | 0.032 | 0.056 | 0.559 |
| Memory CF intercept | ~ | Hypertension | -0.187 | 0.058 | 0.001 |
| Memory CF intercept | ~ | Cardiovascular disease | -0.031 | 0.062 | 0.621 |
| Memory CF intercept | ~ | Diabetes | -0.046 | 0.102 | 0.657 |
| Memory CF intercept | ~ | Stroke | -0.080 | 0.149 | 0.593 |
| Crystallized CF intercept | ~ | Sex | -0.037 | 0.085 | 0.665 |
| Crystallized CF intercept | ~ | Age 11 CF | 2.017 | 0.128 | 0.000 |
| Crystallized CF intercept | ~ | Playing games | 0.343 | 0.086 | 0.000 |
| Crystallized CF intercept | ~ | Education | 0.901 | 0.105 | 0.000 |
| Crystallized CF intercept | ~ | Social class | 0.447 | 0.098 | 0.000 |
| Crystallized CF intercept | ~ | Activity score | 0.239 | 0.091 | 0.009 |
| Crystallized CF intercept | ~ | Hypertension | -0.139 | 0.088 | 0.114 |
| Crystallized CF intercept | ~ | Cardiovascular disease | 0.091 | 0.106 | 0.392 |
| Crystallized CF intercept | ~ | Diabetes | -0.323 | 0.206 | 0.118 |
| Crystallized CF intercept | ~ | Stroke | 0.288 | 0.176 | 0.101 |
| Fluid CF intercept | ~~ | Fluid CF intercept | 0.437 | 0.093 | 0.000 |
| Speed CF intercept | ~~ | Speed CF intercept | 0.676 | 0.089 | 0.000 |
| Memory CF intercept | ~~ | Memory CF intercept | 0.212 | 0.044 | 0.000 |
| Crystallized CF intercept | ~~ | Crystallized CF intercept | 1.266 | 0.087 | 0.000 |
| General CF intercept | ~~ | General CF intercept | 0.747 | 0.097 | 0.000 |
| Fluid CF slope | ~~ | Fluid CF slope | 0.012 | 0.018 | 0.488 |
| Speed CF slope | ~~ | Speed CF slope | 0.020 | 0.018 | 0.264 |
| Memory CF slope | ~~ | Memory CF slope | 0.162 | 0.042 | 0.000 |
| Crystallized CF slope | ~~ | Crystallized CF slope | 0.000 | 0.002 | 0.867 |
| General CF slope | ~~ | General CF slope | 0.025 | 0.020 | 0.207 |
| Sex | ~~ | Sex | 0.250 | 0.000 |  |
| Sex | ~~ | Age 11 CF | 0.012 | 0.000 |  |
| Sex | ~~ | Playing games | 0.028 | 0.000 |  |
| Sex | ~~ | Education | -0.007 | 0.000 |  |
| Sex | ~~ | Social class | 0.029 | 0.000 |  |
| Sex | ~~ | Activity score | 0.051 | 0.000 |  |
| Sex | ~~ | Hypertension | -0.007 | 0.000 |  |
| Sex | ~~ | Cardiovascular disease | -0.022 | 0.000 |  |
| Sex | ~~ | Diabetes | -0.008 | 0.000 |  |
| Sex | ~~ | Stroke | 0.000 | 0.000 |  |
| Age 11 CF | ~~ | Age 11 CF | 0.237 | 0.000 |  |
| Age 11 CF | ~~ | Playing games | 0.034 | 0.000 |  |
| Age 11 CF | ~~ | Education | 0.100 | 0.000 |  |
| Age 11 CF | ~~ | Social class | 0.089 | 0.000 |  |
| Age 11 CF | ~~ | Activity score | 0.076 | 0.000 |  |
| Age 11 CF | ~~ | Hypertension | -0.009 | 0.000 |  |
| Age 11 CF | ~~ | Cardiovascular disease | -0.017 | 0.000 |  |
| Age 11 CF | ~~ | Diabetes | -0.015 | 0.000 |  |
| Age 11 CF | ~~ | Stroke | -0.001 | 0.000 |  |
| Playing games | ~~ | Playing games | 0.248 | 0.000 |  |
| Playing games | ~~ | Education | 0.006 | 0.000 |  |
| Playing games | ~~ | Social class | -0.005 | 0.000 |  |
| Playing games | ~~ | Activity score | 0.017 | 0.000 |  |
| Playing games | ~~ | Hypertension | 0.002 | 0.000 |  |
| Playing games | ~~ | Cardiovascular disease | -0.004 | 0.000 |  |
| Playing games | ~~ | Diabetes | 0.001 | 0.000 |  |
| Playing games | ~~ | Stroke | 0.007 | 0.000 |  |
| Education | ~~ | Education | 0.245 | 0.000 |  |
| Education | ~~ | Social class | 0.111 | 0.000 |  |
| Education | ~~ | Activity score | 0.074 | 0.000 |  |
| Education | ~~ | Hypertension | -0.011 | 0.000 |  |
| Education | ~~ | Cardiovascular disease | -0.004 | 0.000 |  |
| Education | ~~ | Diabetes | -0.006 | 0.000 |  |
| Education | ~~ | Stroke | 0.001 | 0.000 |  |
| Social class | ~~ | Social class | 0.246 | 0.000 |  |
| Social class | ~~ | Activity score | 0.082 | 0.000 |  |
| Social class | ~~ | Hypertension | -0.011 | 0.000 |  |
| Social class | ~~ | Cardiovascular disease | -0.003 | 0.000 |  |
| Social class | ~~ | Diabetes | -0.007 | 0.000 |  |
| Social class | ~~ | Stroke | -0.004 | 0.000 |  |
| Activity score | ~~ | Activity score | 0.281 | 0.000 |  |
| Activity score | ~~ | Hypertension | -0.028 | 0.000 |  |
| Activity score | ~~ | Cardiovascular disease | -0.026 | 0.000 |  |
| Activity score | ~~ | Diabetes | -0.021 | 0.000 |  |
| Activity score | ~~ | Stroke | -0.008 | 0.000 |  |
| Hypertension | ~~ | Hypertension | 0.239 | 0.000 |  |
| Hypertension | ~~ | Cardiovascular disease | 0.044 | 0.000 |  |
| Hypertension | ~~ | Diabetes | 0.028 | 0.000 |  |
| Hypertension | ~~ | Stroke | 0.006 | 0.000 |  |
| Cardiovascular disease | ~~ | Cardiovascular disease | 0.177 | 0.000 |  |
| Cardiovascular disease | ~~ | Diabetes | 0.015 | 0.000 |  |
| Cardiovascular disease | ~~ | Stroke | 0.003 | 0.000 |  |
| Diabetes | ~~ | Diabetes | 0.073 | 0.000 |  |
| Diabetes | ~~ | Stroke | 0.006 | 0.000 |  |
| Stroke | ~~ | Stroke | 0.038 | 0.000 |  |
|  |  |  |  |  |  |

| Table S5. Latent growth curve models of general and subdomain cognitive functions, playing games and difference in playing games, and sociodemographic and health covariates. | | | | | |
| --- | --- | --- | --- | --- | --- |
|  |  |  |  |  |  |
| Path | | | Estimate | Std Error | p |
| General factor associations |  |  |  |  |  |
| General CF slope | ~ | Sex | 0.049 | 0.026 | 0.059 |
| General CF slope | ~ | Age 11 CF | -0.020 | 0.027 | 0.458 |
| General CF slope | ~ | Playing games | 0.088 | 0.033 | 0.007 |
| General CF slope | ~ | Difference in playing games | 0.046 | 0.027 | 0.086 |
| General CF slope | ~ | Education | -0.038 | 0.029 | 0.183 |
| General CF slope | ~ | Social class | -0.013 | 0.027 | 0.634 |
| General CF slope | ~ | Activity score | 0.006 | 0.025 | 0.822 |
| General CF slope | ~ | Hypertension | -0.012 | 0.023 | 0.584 |
| General CF slope | ~ | Cardiovascular disease | -0.019 | 0.030 | 0.527 |
| General CF slope | ~ | Diabetes | -0.127 | 0.052 | 0.014 |
| General CF slope | ~ | Stroke | 0.108 | 0.070 | 0.120 |
| General CF intercept | ~ | Sex | -0.203 | 0.068 | 0.003 |
| General CF intercept | ~ | Age 11 CF | 1.270 | 0.118 | 0.000 |
| General CF intercept | ~ | Playing games | 0.308 | 0.065 | 0.000 |
| General CF intercept | ~ | Education | 0.368 | 0.081 | 0.000 |
| General CF intercept | ~ | Social class | 0.320 | 0.075 | 0.000 |
| General CF intercept | ~ | Activity score | 0.062 | 0.069 | 0.369 |
| General CF intercept | ~ | Hypertension | -0.154 | 0.057 | 0.007 |
| General CF intercept | ~ | Cardiovascular disease | -0.059 | 0.073 | 0.421 |
| General CF intercept | ~ | Diabetes | -0.269 | 0.140 | 0.054 |
| General CF intercept | ~ | Stroke | -0.044 | 0.184 | 0.813 |
| Sex | ~~ | Sex | 0.250 | 0.000 |  |
| Sex | ~~ | Age 11 CF | 0.022 | 0.000 |  |
| Sex | ~~ | Playing games | 0.026 | 0.000 |  |
| Sex | ~~ | Difference in playing games | 0.011 | 0.000 |  |
| Sex | ~~ | Education | 0.004 | 0.000 |  |
| Sex | ~~ | Social class | 0.036 | 0.000 |  |
| Sex | ~~ | Activity score | 0.052 | 0.000 |  |
| Sex | ~~ | Hypertension | -0.002 | 0.000 |  |
| Sex | ~~ | Cardiovascular disease | -0.027 | 0.000 |  |
| Sex | ~~ | Diabetes | -0.011 | 0.000 |  |
| Sex | ~~ | Stroke | -0.003 | 0.000 |  |
| Age 11 CF | ~~ | Age 11 CF | 0.240 | 0.000 |  |
| Age 11 CF | ~~ | Playing games | 0.040 | 0.000 |  |
| Age 11 CF | ~~ | Difference in playing games | 0.014 | 0.000 |  |
| Age 11 CF | ~~ | Education | 0.095 | 0.000 |  |
| Age 11 CF | ~~ | Social class | 0.084 | 0.000 |  |
| Age 11 CF | ~~ | Activity score | 0.062 | 0.000 |  |
| Age 11 CF | ~~ | Hypertension | -0.001 | 0.000 |  |
| Age 11 CF | ~~ | Cardiovascular disease | -0.026 | 0.000 |  |
| Age 11 CF | ~~ | Diabetes | -0.014 | 0.000 |  |
| Age 11 CF | ~~ | Stroke | -0.003 | 0.000 |  |
| Playing games | ~~ | Playing games | 0.247 | 0.000 |  |
| Playing games | ~~ | Difference in playing games | -0.112 | 0.000 |  |
| Playing games | ~~ | Education | 0.013 | 0.000 |  |
| Playing games | ~~ | Social class | 0.002 | 0.000 |  |
| Playing games | ~~ | Activity score | 0.034 | 0.000 |  |
| Playing games | ~~ | Hypertension | 0.006 | 0.000 |  |
| Playing games | ~~ | Cardiovascular disease | 0.000 | 0.000 |  |
| Playing games | ~~ | Diabetes | 0.003 | 0.000 |  |
| Playing games | ~~ | Stroke | 0.003 | 0.000 |  |
| Difference in playing games | ~~ | Difference in playing games | 0.253 | 0.000 |  |
| Difference in playing games | ~~ | Education | 0.000 | 0.000 |  |
| Difference in playing games | ~~ | Social class | 0.001 | 0.000 |  |
| Difference in playing games | ~~ | Activity score | -0.002 | 0.000 |  |
| Difference in playing games | ~~ | Hypertension | -0.009 | 0.000 |  |
| Difference in playing games | ~~ | Cardiovascular disease | -0.004 | 0.000 |  |
| Difference in playing games | ~~ | Diabetes | -0.009 | 0.000 |  |
| Difference in playing games | ~~ | Stroke | -0.006 | 0.000 |  |
| Education | ~~ | Education | 0.244 | 0.000 |  |
| Education | ~~ | Social class | 0.106 | 0.000 |  |
| Education | ~~ | Activity score | 0.073 | 0.000 |  |
| Education | ~~ | Hypertension | -0.010 | 0.000 |  |
| Education | ~~ | Cardiovascular disease | -0.012 | 0.000 |  |
| Education | ~~ | Diabetes | -0.008 | 0.000 |  |
| Education | ~~ | Stroke | -0.002 | 0.000 |  |
| Social class | ~~ | Social class | 0.247 | 0.000 |  |
| Social class | ~~ | Activity score | 0.071 | 0.000 |  |
| Social class | ~~ | Hypertension | -0.003 | 0.000 |  |
| Social class | ~~ | Cardiovascular disease | -0.008 | 0.000 |  |
| Social class | ~~ | Diabetes | -0.005 | 0.000 |  |
| Social class | ~~ | Stroke | -0.004 | 0.000 |  |
| Activity score | ~~ | Activity score | 0.249 | 0.000 |  |
| Activity score | ~~ | Hypertension | -0.020 | 0.000 |  |
| Activity score | ~~ | Cardiovascular disease | -0.024 | 0.000 |  |
| Activity score | ~~ | Diabetes | -0.013 | 0.000 |  |
| Activity score | ~~ | Stroke | -0.004 | 0.000 |  |
| Hypertension | ~~ | Hypertension | 0.236 | 0.000 |  |
| Hypertension | ~~ | Cardiovascular disease | 0.049 | 0.000 |  |
| Hypertension | ~~ | Diabetes | 0.023 | 0.000 |  |
| Hypertension | ~~ | Stroke | 0.012 | 0.000 |  |
| Cardiovascular disease | ~~ | Cardiovascular disease | 0.171 | 0.000 |  |
| Cardiovascular disease | ~~ | Diabetes | 0.017 | 0.000 |  |
| Cardiovascular disease | ~~ | Stroke | 0.002 | 0.000 |  |
| Diabetes | ~~ | Diabetes | 0.064 | 0.000 |  |
| Diabetes | ~~ | Stroke | 0.010 | 0.000 |  |
| Stroke | ~~ | Stroke | 0.029 | 0.000 |  |
| Subdomain associations |  |  |  |  |  |
| Fluid CF slope | ~ | Sex | 0.048 | 0.063 | 0.444 |
| Fluid CF slope | ~ | Age 11 CF | -0.091 | 0.061 | 0.135 |
| Fluid CF slope | ~ | Playing games | 0.053 | 0.054 | 0.330 |
| Fluid CF slope | ~ | Difference in playing games | -0.026 | 0.055 | 0.633 |
| Fluid CF slope | ~ | Education | -0.094 | 0.073 | 0.203 |
| Fluid CF slope | ~ | Social class | 0.017 | 0.050 | 0.726 |
| Fluid CF slope | ~ | Activity score | 0.053 | 0.050 | 0.289 |
| Fluid CF slope | ~ | Hypertension | -0.029 | 0.047 | 0.537 |
| Fluid CF slope | ~ | Cardiovascular disease | -0.030 | 0.058 | 0.601 |
| Fluid CF slope | ~ | Diabetes | -0.042 | 0.091 | 0.646 |
| Fluid CF slope | ~ | Stroke | 0.046 | 0.158 | 0.774 |
| Speed CF slope | ~ | Sex | 0.063 | 0.040 | 0.111 |
| Speed CF slope | ~ | Age 11 CF | -0.001 | 0.042 | 0.973 |
| Speed CF slope | ~ | Playing games | 0.070 | 0.041 | 0.088 |
| Speed CF slope | ~ | Difference in playing games | 0.110 | 0.041 | 0.007 |
| Speed CF slope | ~ | Education | -0.013 | 0.045 | 0.763 |
| Speed CF slope | ~ | Social class | -0.046 | 0.046 | 0.315 |
| Speed CF slope | ~ | Activity score | 0.033 | 0.046 | 0.465 |
| Speed CF slope | ~ | Hypertension | -0.027 | 0.040 | 0.502 |
| Speed CF slope | ~ | Cardiovascular disease | -0.028 | 0.051 | 0.582 |
| Speed CF slope | ~ | Diabetes | -0.141 | 0.085 | 0.097 |
| Speed CF slope | ~ | Stroke | 0.228 | 0.118 | 0.053 |
| Memory CF slope | ~ | Sex | 0.184 | 0.071 | 0.009 |
| Memory CF slope | ~ | Age 11 CF | -0.022 | 0.078 | 0.781 |
| Memory CF slope | ~ | Playing games | 0.204 | 0.076 | 0.007 |
| Memory CF slope | ~ | Difference in playing games | 0.002 | 0.067 | 0.975 |
| Memory CF slope | ~ | Education | 0.027 | 0.076 | 0.719 |
| Memory CF slope | ~ | Social class | -0.040 | 0.079 | 0.615 |
| Memory CF slope | ~ | Activity score | -0.018 | 0.068 | 0.791 |
| Memory CF slope | ~ | Hypertension | 0.032 | 0.065 | 0.622 |
| Memory CF slope | ~ | Cardiovascular disease | 0.024 | 0.079 | 0.763 |
| Memory CF slope | ~ | Diabetes | -0.344 | 0.146 | 0.019 |
| Memory CF slope | ~ | Stroke | 0.289 | 0.167 | 0.083 |
| Crystallised CF slope | ~ | Sex | 0.008 | 0.011 | 0.493 |
| Crystallised CF slope | ~ | Age 11 CF | -0.002 | 0.012 | 0.847 |
| Crystallised CF slope | ~ | Playing games | 0.033 | 0.018 | 0.065 |
| Crystallised CF slope | ~ | Difference in playing games | 0.019 | 0.013 | 0.150 |
| Crystallised CF slope | ~ | Education | -0.028 | 0.019 | 0.152 |
| Crystallised CF slope | ~ | Social class | 0.005 | 0.012 | 0.697 |
| Crystallised CF slope | ~ | Activity score | -0.002 | 0.011 | 0.880 |
| Crystallised CF slope | ~ | Hypertension | -0.005 | 0.010 | 0.625 |
| Crystallised CF slope | ~ | Cardiovascular disease | -0.013 | 0.014 | 0.380 |
| Crystallised CF slope | ~ | Diabetes | -0.039 | 0.030 | 0.195 |
| Crystallised CF slope | ~ | Stroke | 0.015 | 0.031 | 0.623 |
| Fluid CF intercept | ~ | Sex | -0.815 | 0.120 | 0.000 |
| Fluid CF intercept | ~ | Age 11 CF | 1.260 | 0.163 | 0.000 |
| Fluid CF intercept | ~ | Playing games | 0.297 | 0.124 | 0.016 |
| Fluid CF intercept | ~ | Education | 0.440 | 0.155 | 0.004 |
| Fluid CF intercept | ~ | Social class | 0.366 | 0.133 | 0.006 |
| Fluid CF intercept | ~ | Activity score | -0.179 | 0.126 | 0.156 |
| Fluid CF intercept | ~ | Hypertension | -0.133 | 0.123 | 0.278 |
| Fluid CF intercept | ~ | Cardiovascular disease | -0.119 | 0.147 | 0.417 |
| Fluid CF intercept | ~ | Diabetes | -0.537 | 0.232 | 0.020 |
| Fluid CF intercept | ~ | Stroke | -0.220 | 0.362 | 0.544 |
| Speed CF intercept | ~ | Sex | -0.179 | 0.119 | 0.133 |
| Speed CF intercept | ~ | Age 11 CF | 1.116 | 0.145 | 0.000 |
| Speed CF intercept | ~ | Playing games | 0.530 | 0.124 | 0.000 |
| Speed CF intercept | ~ | Education | 0.042 | 0.152 | 0.783 |
| Speed CF intercept | ~ | Social class | 0.574 | 0.129 | 0.000 |
| Speed CF intercept | ~ | Activity score | 0.059 | 0.133 | 0.657 |
| Speed CF intercept | ~ | Hypertension | -0.211 | 0.123 | 0.087 |
| Speed CF intercept | ~ | Cardiovascular disease | -0.256 | 0.148 | 0.083 |
| Speed CF intercept | ~ | Diabetes | -0.364 | 0.278 | 0.191 |
| Speed CF intercept | ~ | Stroke | -0.732 | 0.421 | 0.082 |
| Memory CF intercept | ~ | Sex | -0.066 | 0.054 | 0.225 |
| Memory CF intercept | ~ | Age 11 CF | 0.721 | 0.143 | 0.000 |
| Memory CF intercept | ~ | Playing games | 0.166 | 0.053 | 0.002 |
| Memory CF intercept | ~ | Education | 0.076 | 0.067 | 0.257 |
| Memory CF intercept | ~ | Social class | 0.111 | 0.062 | 0.072 |
| Memory CF intercept | ~ | Activity score | 0.032 | 0.055 | 0.564 |
| Memory CF intercept | ~ | Hypertension | -0.140 | 0.056 | 0.012 |
| Memory CF intercept | ~ | Cardiovascular disease | -0.023 | 0.058 | 0.696 |
| Memory CF intercept | ~ | Diabetes | -0.102 | 0.112 | 0.363 |
| Memory CF intercept | ~ | Stroke | 0.043 | 0.172 | 0.804 |
| Crystallised CF intercept | ~ | Sex | -0.068 | 0.105 | 0.514 |
| Crystallised CF intercept | ~ | Age 11 CF | 2.003 | 0.161 | 0.000 |
| Crystallised CF intercept | ~ | Playing games | 0.365 | 0.105 | 0.000 |
| Crystallised CF intercept | ~ | Education | 0.889 | 0.125 | 0.000 |
| Crystallised CF intercept | ~ | Social class | 0.413 | 0.117 | 0.000 |
| Crystallised CF intercept | ~ | Activity score | 0.223 | 0.126 | 0.076 |
| Crystallised CF intercept | ~ | Hypertension | -0.125 | 0.116 | 0.278 |
| Crystallised CF intercept | ~ | Cardiovascular disease | 0.022 | 0.138 | 0.872 |
| Crystallised CF intercept | ~ | Diabetes | -0.311 | 0.282 | 0.271 |
| Crystallised CF intercept | ~ | Stroke | 0.293 | 0.271 | 0.279 |
| Sex | ~~ | Sex | 0.250 | 0.000 |  |
| Sex | ~~ | Age 11 CF | 0.022 | 0.000 |  |
| Sex | ~~ | Playing games | 0.026 | 0.000 |  |
| Sex | ~~ | Difference in playing games | 0.011 | 0.000 |  |
| Sex | ~~ | Education | 0.004 | 0.000 |  |
| Sex | ~~ | Social class | 0.036 | 0.000 |  |
| Sex | ~~ | Activity score | 0.052 | 0.000 |  |
| Sex | ~~ | Hypertension | -0.002 | 0.000 |  |
| Sex | ~~ | Cardiovascular disease | -0.027 | 0.000 |  |
| Sex | ~~ | Diabetes | -0.011 | 0.000 |  |
| Sex | ~~ | Stroke | -0.003 | 0.000 |  |
| Age 11 CF | ~~ | Age 11 CF | 0.240 | 0.000 |  |
| Age 11 CF | ~~ | Playing games | 0.040 | 0.000 |  |
| Age 11 CF | ~~ | Difference in playing games | 0.014 | 0.000 |  |
| Age 11 CF | ~~ | Education | 0.095 | 0.000 |  |
| Age 11 CF | ~~ | Social class | 0.084 | 0.000 |  |
| Age 11 CF | ~~ | Activity score | 0.062 | 0.000 |  |
| Age 11 CF | ~~ | Hypertension | -0.001 | 0.000 |  |
| Age 11 CF | ~~ | Cardiovascular disease | -0.026 | 0.000 |  |
| Age 11 CF | ~~ | Diabetes | -0.014 | 0.000 |  |
| Age 11 CF | ~~ | Stroke | -0.003 | 0.000 |  |
| Playing games | ~~ | Playing games | 0.247 | 0.000 |  |
| Playing games | ~~ | Difference in playing games | -0.112 | 0.000 |  |
| Playing games | ~~ | Education | 0.013 | 0.000 |  |
| Playing games | ~~ | Social class | 0.002 | 0.000 |  |
| Playing games | ~~ | Activity score | 0.034 | 0.000 |  |
| Playing games | ~~ | Hypertension | 0.006 | 0.000 |  |
| Playing games | ~~ | Cardiovascular disease | 0.000 | 0.000 |  |
| Playing games | ~~ | Diabetes | 0.003 | 0.000 |  |
| Playing games | ~~ | Stroke | 0.003 | 0.000 |  |
| Difference in playing games | ~~ | Difference in playing games | 0.253 | 0.000 |  |
| Difference in playing games | ~~ | Education | 0.000 | 0.000 |  |
| Difference in playing games | ~~ | Social class | 0.001 | 0.000 |  |
| Difference in playing games | ~~ | Activity score | -0.002 | 0.000 |  |
| Difference in playing games | ~~ | Hypertension | -0.009 | 0.000 |  |
| Difference in playing games | ~~ | Cardiovascular disease | -0.004 | 0.000 |  |
| Difference in playing games | ~~ | Diabetes | -0.009 | 0.000 |  |
| Difference in playing games | ~~ | Stroke | -0.006 | 0.000 |  |
| Education | ~~ | Education | 0.244 | 0.000 |  |
| Education | ~~ | Social class | 0.106 | 0.000 |  |
| Education | ~~ | Activity score | 0.073 | 0.000 |  |
| Education | ~~ | Hypertension | -0.010 | 0.000 |  |
| Education | ~~ | Cardiovascular disease | -0.012 | 0.000 |  |
| Education | ~~ | Diabetes | -0.008 | 0.000 |  |
| Education | ~~ | Stroke | -0.002 | 0.000 |  |
| Social class | ~~ | Social class | 0.247 | 0.000 |  |
| Social class | ~~ | Activity score | 0.071 | 0.000 |  |
| Social class | ~~ | Hypertension | -0.003 | 0.000 |  |
| Social class | ~~ | Cardiovascular disease | -0.008 | 0.000 |  |
| Social class | ~~ | Diabetes | -0.005 | 0.000 |  |
| Social class | ~~ | Stroke | -0.004 | 0.000 |  |
| Activity score | ~~ | Activity score | 0.249 | 0.000 |  |
| Activity score | ~~ | Hypertension | -0.020 | 0.000 |  |
| Activity score | ~~ | Cardiovascular disease | -0.024 | 0.000 |  |
| Activity score | ~~ | Diabetes | -0.013 | 0.000 |  |
| Activity score | ~~ | Stroke | -0.004 | 0.000 |  |
| Hypertension | ~~ | Hypertension | 0.236 | 0.000 |  |
| Hypertension | ~~ | Cardiovascular disease | 0.049 | 0.000 |  |
| Hypertension | ~~ | Diabetes | 0.023 | 0.000 |  |
| Hypertension | ~~ | Stroke | 0.012 | 0.000 |  |
| Cardiovascular disease | ~~ | Cardiovascular disease | 0.171 | 0.000 |  |
| Cardiovascular disease | ~~ | Diabetes | 0.017 | 0.000 |  |
| Cardiovascular disease | ~~ | Stroke | 0.002 | 0.000 |  |
| Diabetes | ~~ | Diabetes | 0.064 | 0.000 |  |
| Diabetes | ~~ | Stroke | 0.010 | 0.000 |  |
| Stroke | ~~ | Stroke | 0.029 | 0.000 |  |
|  |  |  |  |  |  |

| Table S6. Latent growth curve models of general and subdomain cognitive functions, playing games and increases in playing games, and sociodemographic and health covariates. | | | | | |
| --- | --- | --- | --- | --- | --- |
|  |  |  |  |  |  |
| Path | | | Estimate | Std Error | p |
| General factor associations | |  |  |  |  |
| General CF slope | ~ | Sex | 0.052 | 0.026 | 0.047 |
| General CF slope | ~ | Age 11 CF | -0.017 | 0.027 | 0.520 |
| General CF slope | ~ | Playing games | 0.084 | 0.033 | 0.010 |
| General CF slope | ~ | Increased games playing | 0.041 | 0.028 | 0.134 |
| General CF slope | ~ | Education | -0.036 | 0.029 | 0.214 |
| General CF slope | ~ | Social class | -0.013 | 0.027 | 0.639 |
| General CF slope | ~ | Activity score | 0.002 | 0.025 | 0.932 |
| General CF slope | ~ | Hypertension | -0.013 | 0.023 | 0.566 |
| General CF slope | ~ | Cardiovascular disease | -0.016 | 0.030 | 0.595 |
| General CF slope | ~ | Diabetes | -0.132 | 0.052 | 0.011 |
| General CF slope | ~ | Stroke | 0.105 | 0.070 | 0.131 |
| General CF intercept | ~ | Sex | -0.202 | 0.068 | 0.003 |
| General CF intercept | ~ | Age 11 CF | 1.268 | 0.118 | 0.000 |
| General CF intercept | ~ | Playing games | 0.307 | 0.064 | 0.000 |
| General CF intercept | ~ | Education | 0.368 | 0.081 | 0.000 |
| General CF intercept | ~ | Social class | 0.319 | 0.074 | 0.000 |
| General CF intercept | ~ | Activity score | 0.062 | 0.069 | 0.367 |
| General CF intercept | ~ | Hypertension | -0.154 | 0.057 | 0.007 |
| General CF intercept | ~ | Cardiovascular disease | -0.059 | 0.073 | 0.419 |
| General CF intercept | ~ | Diabetes | -0.267 | 0.140 | 0.056 |
| General CF intercept | ~ | Stroke | -0.044 | 0.184 | 0.811 |
| Sex | ~~ | Sex | 0.250 | 0.000 |  |
| Sex | ~~ | Age 11 CF | 0.022 | 0.000 |  |
| Sex | ~~ | Playing games | 0.026 | 0.000 |  |
| Sex | ~~ | Increased games playing | 0.002 | 0.000 |  |
| Sex | ~~ | Education | 0.004 | 0.000 |  |
| Sex | ~~ | Social class | 0.036 | 0.000 |  |
| Sex | ~~ | Activity score | 0.052 | 0.000 |  |
| Sex | ~~ | Hypertension | -0.002 | 0.000 |  |
| Sex | ~~ | Cardiovascular disease | -0.027 | 0.000 |  |
| Sex | ~~ | Diabetes | -0.011 | 0.000 |  |
| Sex | ~~ | Stroke | -0.003 | 0.000 |  |
| Age 11 CF | ~~ | Age 11 CF | 0.240 | 0.000 |  |
| Age 11 CF | ~~ | Playing games | 0.040 | 0.000 |  |
| Age 11 CF | ~~ | Increased games playing | 0.000 | 0.000 |  |
| Age 11 CF | ~~ | Education | 0.095 | 0.000 |  |
| Age 11 CF | ~~ | Social class | 0.084 | 0.000 |  |
| Age 11 CF | ~~ | Activity score | 0.062 | 0.000 |  |
| Age 11 CF | ~~ | Hypertension | -0.001 | 0.000 |  |
| Age 11 CF | ~~ | Cardiovascular disease | -0.026 | 0.000 |  |
| Age 11 CF | ~~ | Diabetes | -0.014 | 0.000 |  |
| Age 11 CF | ~~ | Stroke | -0.003 | 0.000 |  |
| Playing games | ~~ | Playing games | 0.247 | 0.000 |  |
| Playing games | ~~ | Increased games playing | -0.093 | 0.000 |  |
| Playing games | ~~ | Education | 0.013 | 0.000 |  |
| Playing games | ~~ | Social class | 0.002 | 0.000 |  |
| Playing games | ~~ | Activity score | 0.034 | 0.000 |  |
| Playing games | ~~ | Hypertension | 0.006 | 0.000 |  |
| Playing games | ~~ | Cardiovascular disease | 0.000 | 0.000 |  |
| Playing games | ~~ | Diabetes | 0.003 | 0.000 |  |
| Playing games | ~~ | Stroke | 0.003 | 0.000 |  |
| Increased games playing | ~~ | Increased games playing | 0.254 | 0.000 |  |
| Increased games playing | ~~ | Education | -0.015 | 0.000 |  |
| Increased games playing | ~~ | Social class | -0.010 | 0.000 |  |
| Increased games playing | ~~ | Activity score | 0.009 | 0.000 |  |
| Increased games playing | ~~ | Hypertension | -0.006 | 0.000 |  |
| Increased games playing | ~~ | Cardiovascular disease | -0.012 | 0.000 |  |
| Increased games playing | ~~ | Diabetes | -0.005 | 0.000 |  |
| Increased games playing | ~~ | Stroke | -0.001 | 0.000 |  |
| Education | ~~ | Education | 0.244 | 0.000 |  |
| Education | ~~ | Social class | 0.106 | 0.000 |  |
| Education | ~~ | Activity score | 0.073 | 0.000 |  |
| Education | ~~ | Hypertension | -0.010 | 0.000 |  |
| Education | ~~ | Cardiovascular disease | -0.012 | 0.000 |  |
| Education | ~~ | Diabetes | -0.008 | 0.000 |  |
| Education | ~~ | Stroke | -0.002 | 0.000 |  |
| Social class | ~~ | Social class | 0.247 | 0.000 |  |
| Social class | ~~ | Activity score | 0.071 | 0.000 |  |
| Social class | ~~ | Hypertension | -0.003 | 0.000 |  |
| Social class | ~~ | Cardiovascular disease | -0.008 | 0.000 |  |
| Social class | ~~ | Diabetes | -0.005 | 0.000 |  |
| Social class | ~~ | Stroke | -0.004 | 0.000 |  |
| Activity score | ~~ | Activity score | 0.249 | 0.000 |  |
| Activity score | ~~ | Hypertension | -0.020 | 0.000 |  |
| Activity score | ~~ | Cardiovascular disease | -0.024 | 0.000 |  |
| Activity score | ~~ | Diabetes | -0.013 | 0.000 |  |
| Activity score | ~~ | Stroke | -0.004 | 0.000 |  |
| Hypertension | ~~ | Hypertension | 0.236 | 0.000 |  |
| Hypertension | ~~ | Cardiovascular disease | 0.049 | 0.000 |  |
| Hypertension | ~~ | Diabetes | 0.023 | 0.000 |  |
| Hypertension | ~~ | Stroke | 0.012 | 0.000 |  |
| Cardiovascular disease | ~~ | Cardiovascular disease | 0.171 | 0.000 |  |
| Cardiovascular disease | ~~ | Diabetes | 0.017 | 0.000 |  |
| Cardiovascular disease | ~~ | Stroke | 0.002 | 0.000 |  |
| Diabetes | ~~ | Diabetes | 0.064 | 0.000 |  |
| Diabetes | ~~ | Stroke | 0.010 | 0.000 |  |
| Stroke | ~~ | Stroke | 0.029 | 0.000 |  |
| Subdomain associations |  |  |  |  |  |
| Fluid CF slope | ~ | Sex | 0.040 | 0.059 | 0.501 |
| Fluid CF slope | ~ | Age 11 CF | -0.092 | 0.060 | 0.124 |
| Fluid CF slope | ~ | Playing games | 0.073 | 0.055 | 0.186 |
| Fluid CF slope | ~ | Increased games playing | 0.021 | 0.046 | 0.651 |
| Fluid CF slope | ~ | Education | -0.089 | 0.071 | 0.214 |
| Fluid CF slope | ~ | Social class | 0.017 | 0.047 | 0.717 |
| Fluid CF slope | ~ | Activity score | 0.045 | 0.048 | 0.348 |
| Fluid CF slope | ~ | Hypertension | -0.029 | 0.044 | 0.512 |
| Fluid CF slope | ~ | Cardiovascular disease | -0.026 | 0.056 | 0.641 |
| Fluid CF slope | ~ | Diabetes | -0.039 | 0.086 | 0.653 |
| Fluid CF slope | ~ | Stroke | 0.042 | 0.152 | 0.781 |
| Speed CF slope | ~ | Sex | 0.071 | 0.040 | 0.077 |
| Speed CF slope | ~ | Age 11 CF | 0.009 | 0.042 | 0.839 |
| Speed CF slope | ~ | Playing games | 0.043 | 0.041 | 0.296 |
| Speed CF slope | ~ | Increased games playing | 0.064 | 0.043 | 0.139 |
| Speed CF slope | ~ | Education | -0.012 | 0.046 | 0.795 |
| Speed CF slope | ~ | Social class | -0.048 | 0.047 | 0.300 |
| Speed CF slope | ~ | Activity score | 0.031 | 0.046 | 0.504 |
| Speed CF slope | ~ | Hypertension | -0.029 | 0.041 | 0.478 |
| Speed CF slope | ~ | Cardiovascular disease | -0.022 | 0.052 | 0.673 |
| Speed CF slope | ~ | Diabetes | -0.151 | 0.086 | 0.078 |
| Speed CF slope | ~ | Stroke | 0.221 | 0.122 | 0.070 |
| Memory CF slope | ~ | Sex | 0.184 | 0.071 | 0.010 |
| Memory CF slope | ~ | Age 11 CF | -0.026 | 0.077 | 0.732 |
| Memory CF slope | ~ | Playing games | 0.222 | 0.073 | 0.002 |
| Memory CF slope | ~ | Increased games playing | 0.046 | 0.071 | 0.522 |
| Memory CF slope | ~ | Education | 0.031 | 0.076 | 0.681 |
| Memory CF slope | ~ | Social class | -0.038 | 0.079 | 0.631 |
| Memory CF slope | ~ | Activity score | -0.024 | 0.068 | 0.727 |
| Memory CF slope | ~ | Hypertension | 0.032 | 0.065 | 0.621 |
| Memory CF slope | ~ | Cardiovascular disease | 0.025 | 0.079 | 0.751 |
| Memory CF slope | ~ | Diabetes | -0.345 | 0.147 | 0.019 |
| Memory CF slope | ~ | Stroke | 0.283 | 0.167 | 0.090 |
| Crystallized CF slope | ~ | Sex | 0.009 | 0.011 | 0.436 |
| Crystallized CF slope | ~ | Age 11 CF | -0.001 | 0.012 | 0.960 |
| Crystallized CF slope | ~ | Playing games | 0.029 | 0.016 | 0.073 |
| Crystallized CF slope | ~ | Increased games playing | 0.011 | 0.011 | 0.308 |
| Crystallized CF slope | ~ | Education | -0.028 | 0.019 | 0.153 |
| Crystallized CF slope | ~ | Social class | 0.005 | 0.012 | 0.712 |
| Crystallized CF slope | ~ | Activity score | -0.002 | 0.012 | 0.842 |
| Crystallized CF slope | ~ | Hypertension | -0.006 | 0.011 | 0.607 |
| Crystallized CF slope | ~ | Cardiovascular disease | -0.012 | 0.015 | 0.414 |
| Crystallized CF slope | ~ | Diabetes | -0.041 | 0.031 | 0.184 |
| Crystallized CF slope | ~ | Stroke | 0.014 | 0.032 | 0.663 |
| Fluid CF intercept | ~ | Sex | -0.811 | 0.121 | 0.000 |
| Fluid CF intercept | ~ | Age 11 CF | 1.259 | 0.162 | 0.000 |
| Fluid CF intercept | ~ | Playing games | 0.295 | 0.124 | 0.017 |
| Fluid CF intercept | ~ | Education | 0.441 | 0.154 | 0.004 |
| Fluid CF intercept | ~ | Social class | 0.368 | 0.133 | 0.006 |
| Fluid CF intercept | ~ | Activity score | -0.176 | 0.126 | 0.161 |
| Fluid CF intercept | ~ | Hypertension | -0.131 | 0.122 | 0.281 |
| Fluid CF intercept | ~ | Cardiovascular disease | -0.121 | 0.146 | 0.410 |
| Fluid CF intercept | ~ | Diabetes | -0.536 | 0.230 | 0.020 |
| Fluid CF intercept | ~ | Stroke | -0.216 | 0.359 | 0.547 |
| Speed CF intercept | ~ | Sex | -0.179 | 0.120 | 0.135 |
| Speed CF intercept | ~ | Age 11 CF | 1.115 | 0.145 | 0.000 |
| Speed CF intercept | ~ | Playing games | 0.529 | 0.124 | 0.000 |
| Speed CF intercept | ~ | Education | 0.043 | 0.152 | 0.777 |
| Speed CF intercept | ~ | Social class | 0.575 | 0.129 | 0.000 |
| Speed CF intercept | ~ | Activity score | 0.058 | 0.132 | 0.659 |
| Speed CF intercept | ~ | Hypertension | -0.211 | 0.123 | 0.086 |
| Speed CF intercept | ~ | Cardiovascular disease | -0.258 | 0.148 | 0.081 |
| Speed CF intercept | ~ | Diabetes | -0.362 | 0.278 | 0.193 |
| Speed CF intercept | ~ | Stroke | -0.734 | 0.420 | 0.080 |
| Memory CF intercept | ~ | Sex | -0.067 | 0.054 | 0.221 |
| Memory CF intercept | ~ | Age 11 CF | 0.723 | 0.144 | 0.000 |
| Memory CF intercept | ~ | Playing games | 0.165 | 0.053 | 0.002 |
| Memory CF intercept | ~ | Education | 0.076 | 0.067 | 0.259 |
| Memory CF intercept | ~ | Social class | 0.112 | 0.062 | 0.072 |
| Memory CF intercept | ~ | Activity score | 0.032 | 0.055 | 0.560 |
| Memory CF intercept | ~ | Hypertension | -0.141 | 0.056 | 0.013 |
| Memory CF intercept | ~ | Cardiovascular disease | -0.023 | 0.058 | 0.698 |
| Memory CF intercept | ~ | Diabetes | -0.101 | 0.112 | 0.367 |
| Memory CF intercept | ~ | Stroke | 0.043 | 0.172 | 0.802 |
| Crystallized CF intercept | ~ | Sex | -0.068 | 0.105 | 0.514 |
| Crystallized CF intercept | ~ | Age 11 CF | 2.003 | 0.161 | 0.000 |
| Crystallized CF intercept | ~ | Playing games | 0.365 | 0.105 | 0.001 |
| Crystallized CF intercept | ~ | Education | 0.889 | 0.125 | 0.000 |
| Crystallized CF intercept | ~ | Social class | 0.413 | 0.117 | 0.000 |
| Crystallized CF intercept | ~ | Activity score | 0.223 | 0.126 | 0.077 |
| Crystallized CF intercept | ~ | Hypertension | -0.125 | 0.115 | 0.277 |
| Crystallized CF intercept | ~ | Cardiovascular disease | 0.022 | 0.138 | 0.871 |
| Crystallized CF intercept | ~ | Diabetes | -0.310 | 0.283 | 0.273 |
| Crystallized CF intercept | ~ | Stroke | 0.293 | 0.271 | 0.281 |
| Sex | ~~ | Sex | 0.250 | 0.000 |  |
| Sex | ~~ | Age 11 CF | 0.022 | 0.000 |  |
| Sex | ~~ | Playing games | 0.026 | 0.000 |  |
| Sex | ~~ | Increased games playing | 0.002 | 0.000 |  |
| Sex | ~~ | Education | 0.004 | 0.000 |  |
| Sex | ~~ | Social class | 0.036 | 0.000 |  |
| Sex | ~~ | Activity score | 0.052 | 0.000 |  |
| Sex | ~~ | Hypertension | -0.002 | 0.000 |  |
| Sex | ~~ | Cardiovascular disease | -0.027 | 0.000 |  |
| Sex | ~~ | Diabetes | -0.011 | 0.000 |  |
| Sex | ~~ | Stroke | -0.003 | 0.000 |  |
| Age 11 CF | ~~ | Age 11 CF | 0.240 | 0.000 |  |
| Age 11 CF | ~~ | Playing games | 0.040 | 0.000 |  |
| Age 11 CF | ~~ | Increased games playing | 0.000 | 0.000 |  |
| Age 11 CF | ~~ | Education | 0.095 | 0.000 |  |
| Age 11 CF | ~~ | Social class | 0.084 | 0.000 |  |
| Age 11 CF | ~~ | Activity score | 0.062 | 0.000 |  |
| Age 11 CF | ~~ | Hypertension | -0.001 | 0.000 |  |
| Age 11 CF | ~~ | Cardiovascular disease | -0.026 | 0.000 |  |
| Age 11 CF | ~~ | Diabetes | -0.014 | 0.000 |  |
| Age 11 CF | ~~ | Stroke | -0.003 | 0.000 |  |
| Playing games | ~~ | Playing games | 0.247 | 0.000 |  |
| Playing games | ~~ | Increased games playing | -0.093 | 0.000 |  |
| Playing games | ~~ | Education | 0.013 | 0.000 |  |
| Playing games | ~~ | Social class | 0.002 | 0.000 |  |
| Playing games | ~~ | Activity score | 0.034 | 0.000 |  |
| Playing games | ~~ | Hypertension | 0.006 | 0.000 |  |
| Playing games | ~~ | Cardiovascular disease | 0.000 | 0.000 |  |
| Playing games | ~~ | Diabetes | 0.003 | 0.000 |  |
| Playing games | ~~ | Stroke | 0.003 | 0.000 |  |
| Increased games playing | ~~ | Increased games playing | 0.254 | 0.000 |  |
| Increased games playing | ~~ | Education | -0.015 | 0.000 |  |
| Increased games playing | ~~ | Social class | -0.010 | 0.000 |  |
| Increased games playing | ~~ | Activity score | 0.009 | 0.000 |  |
| Increased games playing | ~~ | Hypertension | -0.006 | 0.000 |  |
| Increased games playing | ~~ | Cardiovascular disease | -0.012 | 0.000 |  |
| Increased games playing | ~~ | Diabetes | -0.005 | 0.000 |  |
| Increased games playing | ~~ | Stroke | -0.001 | 0.000 |  |
| Education | ~~ | Education | 0.244 | 0.000 |  |
| Education | ~~ | Social class | 0.106 | 0.000 |  |
| Education | ~~ | Activity score | 0.073 | 0.000 |  |
| Education | ~~ | Hypertension | -0.010 | 0.000 |  |
| Education | ~~ | Cardiovascular disease | -0.012 | 0.000 |  |
| Education | ~~ | Diabetes | -0.008 | 0.000 |  |
| Education | ~~ | Stroke | -0.002 | 0.000 |  |
| Social class | ~~ | Social class | 0.247 | 0.000 |  |
| Social class | ~~ | Activity score | 0.071 | 0.000 |  |
| Social class | ~~ | Hypertension | -0.003 | 0.000 |  |
| Social class | ~~ | Cardiovascular disease | -0.008 | 0.000 |  |
| Social class | ~~ | Diabetes | -0.005 | 0.000 |  |
| Social class | ~~ | Stroke | -0.004 | 0.000 |  |
| Activity score | ~~ | Activity score | 0.249 | 0.000 |  |
| Activity score | ~~ | Hypertension | -0.020 | 0.000 |  |
| Activity score | ~~ | Cardiovascular disease | -0.024 | 0.000 |  |
| Activity score | ~~ | Diabetes | -0.013 | 0.000 |  |
| Activity score | ~~ | Stroke | -0.004 | 0.000 |  |
| Hypertension | ~~ | Hypertension | 0.236 | 0.000 |  |
| Hypertension | ~~ | Cardiovascular disease | 0.049 | 0.000 |  |
| Hypertension | ~~ | Diabetes | 0.023 | 0.000 |  |
| Hypertension | ~~ | Stroke | 0.012 | 0.000 |  |
| Cardiovascular disease | ~~ | Cardiovascular disease | 0.171 | 0.000 |  |
| Cardiovascular disease | ~~ | Diabetes | 0.017 | 0.000 |  |
| Cardiovascular disease | ~~ | Stroke | 0.002 | 0.000 |  |
| Diabetes | ~~ | Diabetes | 0.064 | 0.000 |  |
| Diabetes | ~~ | Stroke | 0.010 | 0.000 |  |
| Stroke | ~~ | Stroke | 0.029 | 0.000 |  |
|  |  |  |  |  |  |

| Table S7. Principal components analysis of socio-intellectual activities. | | |
| --- | --- | --- |
|  |  |  |
| Variable | Loading | Communality |
| Physical activity | 0.29 | 0.09 |
| Visits libraries | 0.39 | 0.15 |
| Reads newspapers/magazines | 0.07 | 0.01 |
| Reads books | 0.42 | 0.18 |
| Writes for pleasure | 0.41 | 0.17 |
| Listens to the radio | 0.40 | 0.16 |
| Visits friends or family | 0.43 | 0.18 |
| Studies course at work or takes evening classes | 0.44 | 0.19 |
| Goes to the cinema or restaurants | 0.58 | 0.33 |
| Goes to sporting events or concerts | 0.59 | 0.35 |
| Goes to the theatre, galleries, or museums | 0.69 | 0.48 |
| Participates in social groups | 0.56 | 0.32 |
| Participates in church or religious activities | 0.40 | 0.16 |
| SS loadings | 2.76 |  |
| Proportion of variance explained | 0.21 |  |

**Supplementary References**

Deary, I. J., Johnson, W., & Starr, J. M. (2010). Are processing speed tasks biomarkers of cognitive aging? *Psychology and aging, 25*(1), 219.

Elias, P., McKnight, A., & Kinshott, G. (1999). SOC 2000: redefining skill: revision of the standard occupational classification.

Ginns, P., & Barrie, S. (2004). Reliability of single-item ratings of quality in higher education: A replication. *Psychological Reports, 95*(3), 1023-1030.

Horn, J. L., & Cattell, R. B. (1967). Age differences in fluid and crystallized intelligence. *Acta psychologica, 26*, 107-129.

Ritchie, S. J., Tucker-Drob, E. M., Cox, S. R., Corley, J., Dykiert, D., Redmond, P., . . . Starr, J. M. (2016). Predictors of ageing-related decline across multiple cognitive functions. *Intelligence, 59*, 115-126.

Taylor, A. M., Pattie, A., & Deary, I. J. (2018). Cohort Profile Update: The Lothian Birth Cohorts of 1921 and 1936. *International Journal of Epidemiology, 47*(4), 1042-1042r. doi:10.1093/ije/dyy022

Verhaeghen, P., & Salthouse, T. A. (1997). Meta-analyses of age–cognition relations in adulthood: Estimates of linear and nonlinear age effects and structural models. *Psychological bulletin, 122*(3), 231.

Wanous, J. P., Reichers, A. E., & Hudy, M. J. (1997). Overall job satisfaction: how good are single-item measures? *Journal of applied psychology, 82*(2), 247.

Waris, O., Jaeggi, S. M., Seitz, A. R., Lehtonen, M., Soveri, A., Lukasik, K. M., . . . Laine, M. (2019). Video gaming and working memory: A large-scale cross-sectional correlative study. *Computers in Human Behavior, 97*, 94-103. doi:<https://doi.org/10.1016/j.chb.2019.03.005>

Wechsler, D. (1945). Wechsler memory scale.

Wechsler, D. (1997). Adult intelligence scale. *New York, 21*.
